# Supplementary material for: Inflammatory macrophages reprogram to immunosuppression by reducing mitochondrial translation
Source: Nat Commun. 2023 Nov 17;14:7471. doi: 10.1038/s41467-023-42277-4 (PMC10656499; doi:10.1038/s41467-023-42277-4)
Supplement: Supplementary file 1 — Supplementary Information [file 41467_2023_42277_MOESM1_ESM.pdf]

# SUPPLEMENTARY INFORMATION

# FOR

# Macrophage reprogramming toward an immunosuppressive phenotype is driven by reduced amino acid-dependent mitochondrial translation

Marlies Cortés[1,*], Agnese Brischetto[1#], MC Martinez-Campanario[1#], Chiara Ninfali[1], Verónica Domínguez[2], Sara Fernández[3], Raquel Celis[4], Anna Esteve-Codina[5], Juan J. Lozano[6], Julia Sidorova[6], Gloria Garrabou[3], Anna-Maria Siegert[7], Carlos Enrich[8], Belén Pintado[2], Manuel Morales-Ruiz[6,8,9], Pedro Castro[3], Juan D. Cañete[4], Antonio Postigo[1,10,11,*]

[1] Group of Gene Regulation in Stem Cells, Cell Plasticity, Differentiation, and Cancer. IDIBAPS, 08036 Barcelona, Spain
[2] National Center of Biotechnology (CSIC-CNB) and Center for Molecular Biology Severo Ochoa (CSIC/UAM-CBMSO) Transgenesis Facility. Higher Research Council (CSIC) and Autonomous University of Madrid (UAM), Cantoblanco, 28049 Madrid, Spain
[3] Medical Intensive Care Unit and Dept. of Internal Medicine. Hospital Clínic of Barcelona, Group of Muscle Research and Mitochondrial Function, IDIBAPS, and CIBERER, 08036 Barcelona, Spain
[4] Arthritis Unit, Dept. of Rheumathology, Hospital Clínic and IDIBAPS, 08036 Barcelona, Spain
[5] National Center for Genomics Analysis and Centre for Genomic Regulation (CNAG-CRG), Barcelona Institute of Science & Technology, and Universitat Pompeu Fabra, 08003 Barcelona, Spain.
[6] Biomedical Research Networking Centers in Digestive and Hepatic Diseases( CIBERehd). Carlos III Health Institute. 08036 Barcelona, Spain
[7] MRC Metabolic Diseases Unit, University of Cambridge Metabolic Research Laboratories, Wellcome Trust-MRC Institute of Metabolic Science, Addenbrooke's Hospital, Cambridge, CB1 0QQ, UK
[8] Dept. of Biomedicine, University of Barcelona School of Medicine and Health Sciences, 08036 Barcelona, Spain
[9] Dept. of Biochemistry and Molecular Genetics, Hospital Clínic of Barcelona and IDIBAPS, 08036 Barcelona, Spain
[10] Molecular Targets Program, Div. of Oncology, Dept. of Medicine, J.G. Brown Cancer Center. Louisville, KY, 40202, USA
[11] ICREA, 08010 Barcelona, Spain

# AG and MCM-C are equal second-authors

* Corresponding authors: M Cortés (mcortesh@recerca.clinic.cat) and A Postigo (idib412@clinic.cat). Group of Gene Regulation in Stem Cells, Cell Plasticity, Differentiation, and Cancer, IDIBAPS, 08036 Barcelona, Spain

Keywords: ZEB1 / EMT / Macrophage / Metformin / Mitochondria / Metabolism / Inflammation / Immunosuppression /

**This Supplementary Information file includes:**

- Supplementary Figures S1 to S6
- Supplementary Tables S1 to S8
- Supplementary Methods
- Supplementary References

## **SUPPLEMENTARY FIGURES**

# Supplementary Figure S1

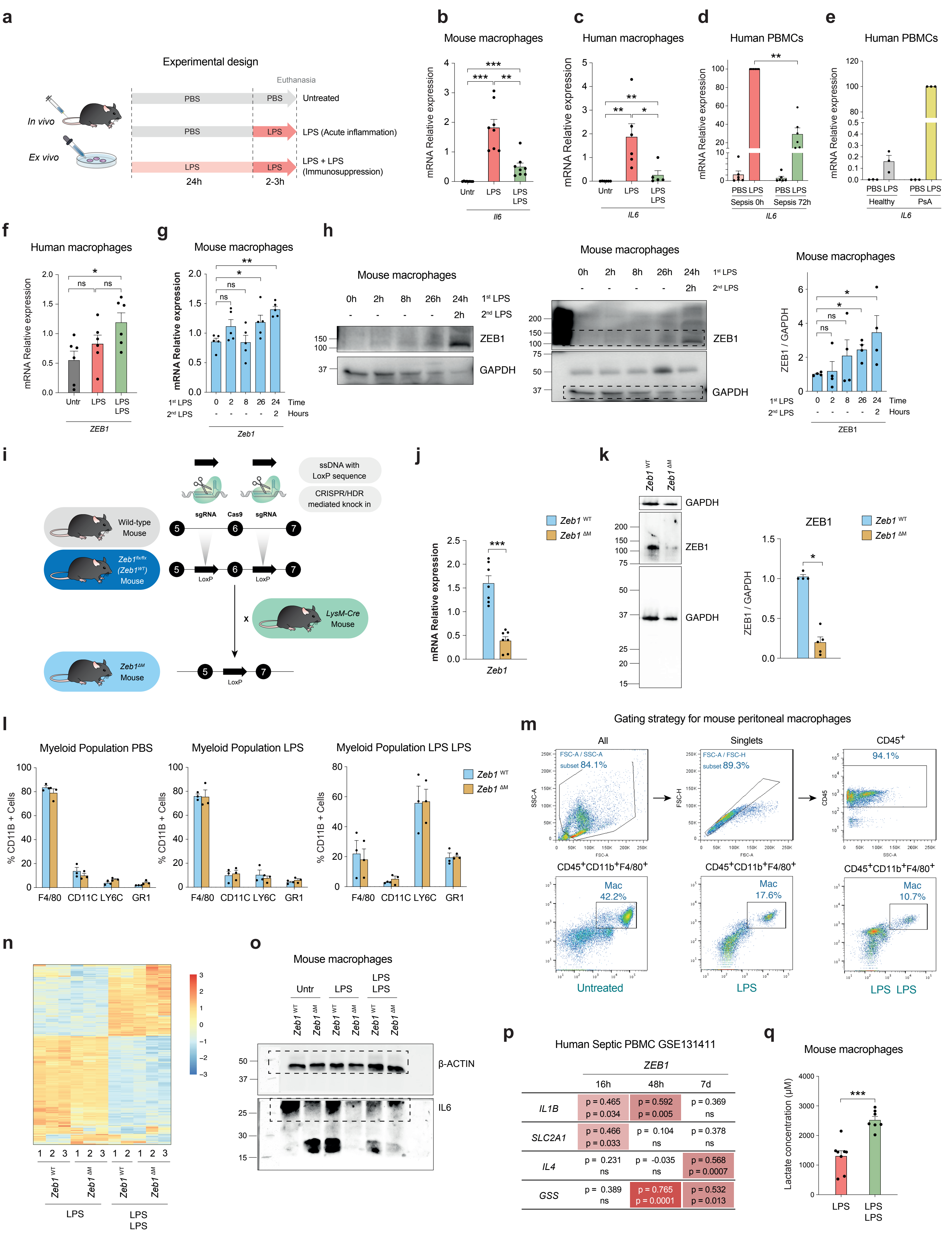

## SUPPLEMENTARY FIGURE S1

**ZEB1 has a dual role that is required for both the induction and resolution of inflammation. (a)** Experimental design: *In vivo* and ex vivo protocols of acute inflammation and immunosuppression. **(b)** *Il6* mRNA levels in peritoneal macrophages from *Zeb1*$^{WT}$ mice either untreated, treated with LPS, or “LPS + LPS” (n=8). with one or two mice per condition. **(c)** *Il6* mRNA levels in human monocyte-derived macrophages (MDM) either untreated, treated with LPS, or LPS+LPS. Data are the average of five independent experiments (n=6,6,5). **(d)** *IL6* mRNA levels in human PBMCs from six septic patients at 0 h and 72 h from the time of ICU admission. **(e)** *IL6* mRNA levels in human PBMCs from three healthy donors and three psoriatic arthritis (PsA) patients that had been either left untreated or treated with LPS. **(f)** *ZEB1* mRNA levels in human MDM either untreated, treated with LPS, or LPS + LPS. Data are the average of six independent experiments (n=6). **(g)** *Zeb1* mRNA levels in *Zeb1*$^{WT}$ peritoneal macrophages either untreated or treated with LPS for different periods or LPS + LPS. Data are the average of three independent experiments (n=5) with a total of 5 mice per condition. **(h)** Cell lysates from *Zeb1*$^{WT}$ peritoneal macrophages either untreated or treated with LPS for different periods or LPS + LPS were blotted for ZEB1 [home-made, (1), dilution: 1/1000] and GAPDH (1E6D9, 1/15000) as loading control. Cropped and uncropped blots are shown. Quantification of ZEB1 levels relative to GAPDH levels in four experiments as in the left panel (n=4). (**i**) A schematic depicting the generation of *Zeb1*$^{\Delta M}$ mouse is shown. **(j)** ZEB1 Relative mRNA expression in sorted peritoneal macrophages from *Zeb1*$^{WT}$ and *Zeb1*$^{\Delta M}$ mice (n=7) in at least 3 independent experiments. **(k)** As in (I), but cell lysates were blotted with ZEB1 [home-made (1), 1/1000] and GAPDH (1E6D9, 1/15000). Blots shown are representative of at least three experiments. **(l)** Distribution of myeloid cells subpopulations in the peritoneal cavity of *Zeb1*$^{WT}$ and *Zeb1*$^{\Delta M}$ mice treated for 2 h with PBS, LPS, or LPS + LPS (n=3). **(m)** Gating strategy used for the sorting and analysis of peritoneal macrophages by FACS. **(n)** Heatmap with the regularized log-transformed expression of the top 500 differentially expressed genes. **(o)** Uncropped blots for Fig. 1B. **(p)** The correlation ($\rho$ Pearson's coefficient with p-value) between the expression of *ZEB1* and either *IL1B*, *SLC2A1*, *IL4*, or *GSS* in human PBMCs from septic patients at 16 h, 48 h and 7 days post-admission at the ICU was investigated in a published array [GSE131411, (2)]. **(q)** Lactate levels in the supernatant of peritoneal macrophages from *Zeb1*$^{WT}$ mice subjected *in vivo* to LPS or LPS + LPS. Data are the average of two independent experiments (n=8,7), each with 3-4 mice per condition. Graph bars in Figure represent mean values +/- SEM with two-tailed unpaired Mann-Whitney test. $p \leq 0.001$ (***), $p \leq 0.01$ (**) or $p \leq 0.05$ (*) levels, or non-significant (ns) for values of $p > 0.05$. Raw data along p values for statistical analyses are included in the Source Data file.

# Supplementary Figure S2

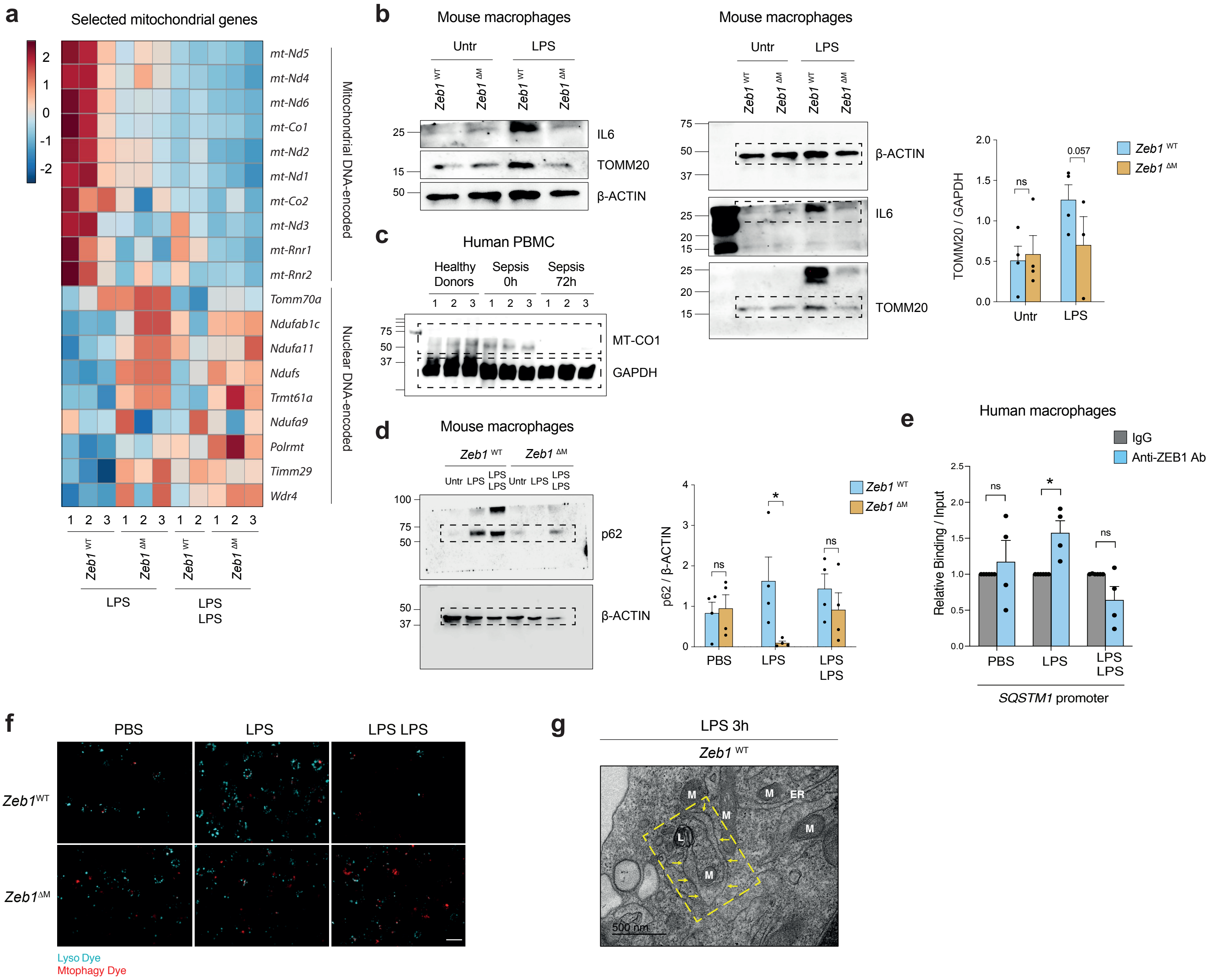

## SUPPLEMENTARY FIGURE S2

**ZEB1 increases mitochondrial content and autophagy during inflammation. (a)** Heatmap of the expression of selected differentially expressed mitochondrial-related genes (DNA- and nuclear-encoded) in peritoneal macrophages from *Zeb1*$^{WT}$ and *Zeb1*$^{\Delta M}$ treated with LPS and LPS + LPS. **(b)** Cell lysates from *Zeb1*$^{WT}$ and *Zeb1*$^{\Delta M}$ macrophages either untreated or treated with LPS were blotted for IL6 (IT1G2, 1/200), TOMM20 (ab56783, 1/200), and β-ACTIN (C4 1/400). Blots are representative of three independent experiments. Uncropped blots are shown on the left. **(c)** Uncropped blots for Fig. 1H. **(d)** *Left panel:* Uncropped blots for Fig. 1L. *Right panel*: quantification of p62 protein levels relative to β-ACTIN protein levels in four independent experiments. **(e)** Binding of ZEB1 to the human *SQSTM1* promoter in human CSF1-induced MDM either untreated, treated with LPS, or LPS+LPS was determined by ChIP. Data are the average of four independent experiments. **(f)** Peritoneal macrophages from *Zeb1*$^{WT}$ and *Zeb1*$^{\Delta M}$ mice treated for 3 h with PBS, LPS, or LPS + LPS were stained with Mtphagy Dye and Lyso Dye for the formation of autophagosome and phagosome fused to lysosomes. **(g)** Representative TEM caption of *Zeb1*$^{WT}$ macrophages treated *in vitro* with LPS for 3 h as in Fig. 1I. The yellow square frames an autolysosome (surrounded with arrows) in close contact with a multilamellar lysosome, in a process of wrapping a mitochondria together with cytosolic material. L: lysosome; M: mitochondria; ER: endoplasmic reticulum. Scale bar: 500 nm. Graph bars in Figure represent mean values +/- SEM with two-tailed unpaired Mann-Whitney test. $p \leq 0.001$ (***), $p \leq 0.01$ (**) or $p \leq 0.05$ (*) levels, or non-significant (ns) for values of $p > 0.05$. Raw data along p values for statistical analyses are included in the Source Data file.

## Supplementary Figure S3

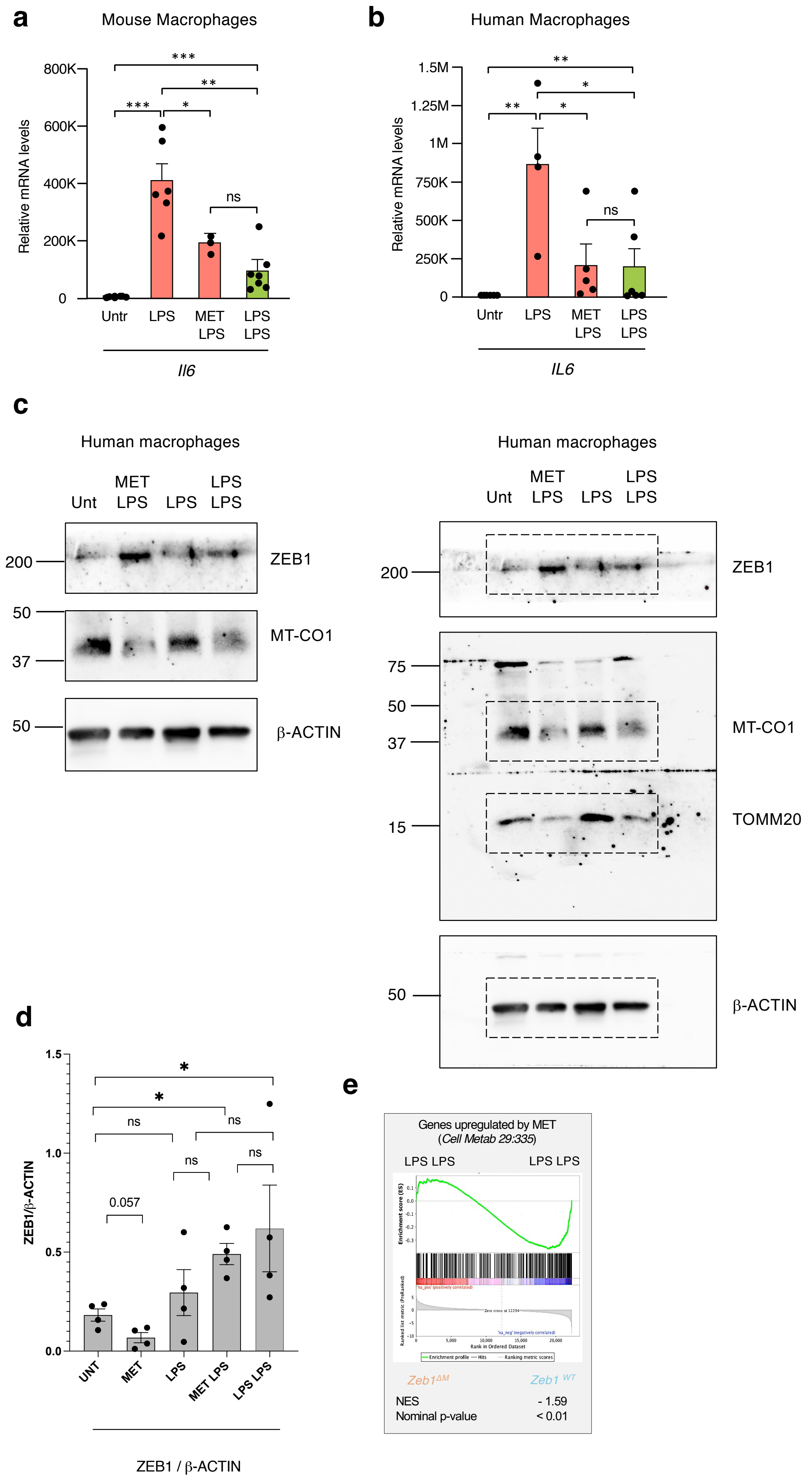

## SUPPLEMENTARY FIGURE S3

**Metformin depends on ZEB1 expression in macrophages for its anti-inflammatory effects. (A)** *Il6* mRNA levels in peritoneal macrophages from *Zeb1*$^{WT}$ either untreated or treated with LPS, metformin + LPS, or LPS + LPS. Data are the average of three independent experiments (n=8,6,3,8). **(B)** *IL6* mRNA levels in human MDM either untreated, or treated with LPS, metformin + LPS, or LPS + LPS. Data are the average of four independent experiments (n=6,4,5,6) **(C)** Lysates of human MDM either untreated, or treated with metformin + LPS, LPS, or LPS + LPS were blotted for MT-CO1 (PA1317-1, 1/400) and ZEB1 (H3, 1/200) protein, and β-ACTIN (C4, 1/400). *Left panel*: Blots are representative of four independent experiments. *Right panel*: uncropped blots corresponding to those on the left. **(D)** Quantification of ZEB1 protein levels relative to β-ACTIN protein levels in four experiments as in the left panel. **(E)** GSEA for the expression of a metformin-associated signature in published array GSE98731 (3) for *Zeb1*$^{WT}$ and *Zeb1*$^{\Delta M}$ macrophages treated with LPS+LPS. Graph bars in Figure represent mean values +/- SEM with two-tailed unpaired Mann-Whitney test. $p \leq 0.001$ (***), $p \leq 0.01$ (**) or $p \leq 0.05$ (*) levels, or non-significant (ns) for values of $p > 0.05$. Raw data along p values for statistical analyses are included in the Source Data file.

# Supplementary Figure S4

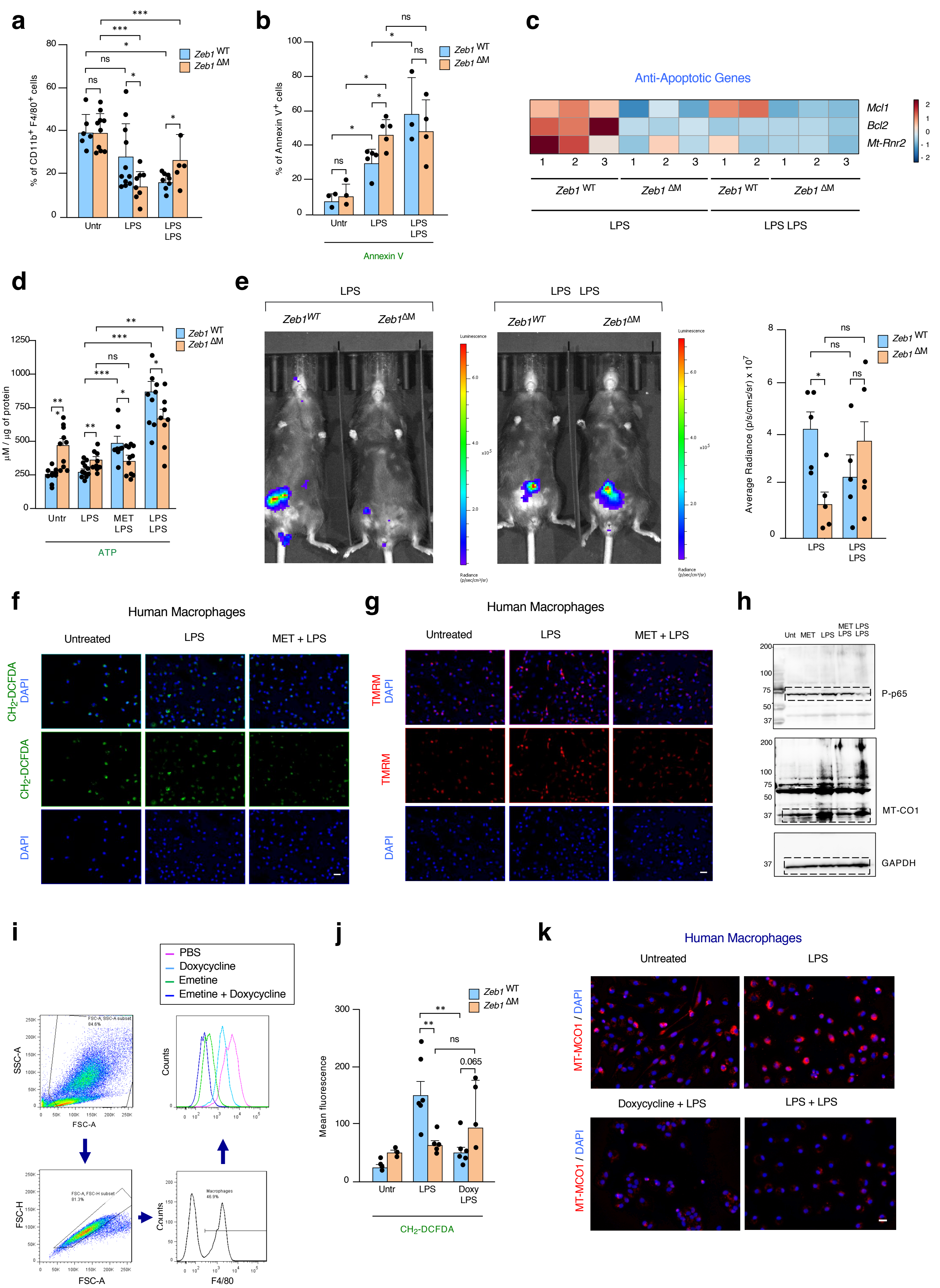

## SUPLEMENTARY FIGURE S4

**ZEB1 mediates the immunosuppression-mimicking effect of metformin by reducing mitochondrial content and ROS levels. (A)** Percentage of CD11b$^+$ F4/80$^+$ macrophages in the peritoneal cavity of *Zeb1*$^{WT}$ and *Zeb1*$^{\Delta M}$ mice either untreated or treated with LPS or LPS + LPS (n=6,10,9,8,8,6 mice). Data are the average of a total of 6-9 mice for each genotype and condition. **(B)** Average of the median fluorescence intensity for Annexin V of *Zeb1*$^{WT}$ and *Zeb1*$^{\Delta M}$ mice either untreated or treated with LPS or LPS + LPS (n=3,3,5,5,3,4 mice). Data was obtained in three independent experiments with one or two mice for each genotype and condition. **(C)** Heatmap of the expression of selected differentially expressed anti-apoptotic genes in peritoneal macrophages from *Zeb1*$^{WT}$ and *Zeb1*$^{\Delta M}$ mice treated with LPS or LPS+LPS. **(D)** ATP levels in *Zeb1*$^{WT}$ and *Zeb1*$^{\Delta M}$ peritoneal macrophages either untreated, or treated with LPS, metformin + LPS, or LPS + LPS. Data originated from 8-10 mice per genotype (n=10,10,10,9,9,10,9,8). **(E)** *In vivo* ROS production in *Zeb1*$^{WT}$ and *Zeb1*$^{\Delta M}$ mice treated with LPS or LPS + LPS and injected i.p. with the L-012 probe. *Left panel*: The pictures of the bioluminescence signal shown are representative of five mice per genotype and condition. *Right panel*: quantification of average radiance (p/s/cm≤/sr) x $10^7$ (n=5). **(F)** Immunocytochemistry of human MDM either untreated or treated *in vitro* with LPS or metformin + LPS stained for $CH_2$-DCFDA along with DAPI. Captions shown are representative of two independent experiments. Scale bar: 25 µm. **(G)** As in (F), but human MDM were stained for TMRM. Pictures are representative of two independent experiments. **(H)** Uncropped blots for Fig. 4F. **(I)** The FACS gating strategy and representative plots used to assess mitochondrial translation in doxycycline and Emetine-treated samples. **(J)** As in Fig. 4J, quantification of $CH_2$-DCFDA staining in macrophages isolated from *Zeb1*$^{WT}$ and *Zeb1*$^{\Delta M}$ mice either untreated or treated with LPS or “doxycycline + LPS”. Bars are the average of the mean fluorescence intensity of two independent experiments with a total of 4-6 mice for each genotype and condition (n=4,3,6,5,6,6 mice). **(K)** Immunocytochemistry for MT-CO1 (PA1317-1, 1/100) staining along with DAPI of human MDM either untreated or treated with LPS, “doxycycline + LPS”, or LPS + LPS. Scale bar: 10 µm. Graph bars in Figure represent mean values +/- SEM with two-tailed unpaired Mann-Whitney test. $p \leq 0.001$ (***), $p \leq 0.01$ (**) or $p \leq 0.05$ (*) levels, or non-significant (ns) for values of $p > 0.05$. Raw data along p values for statistical analyses are included in the Source Data file.

# Supplementary Figure S5

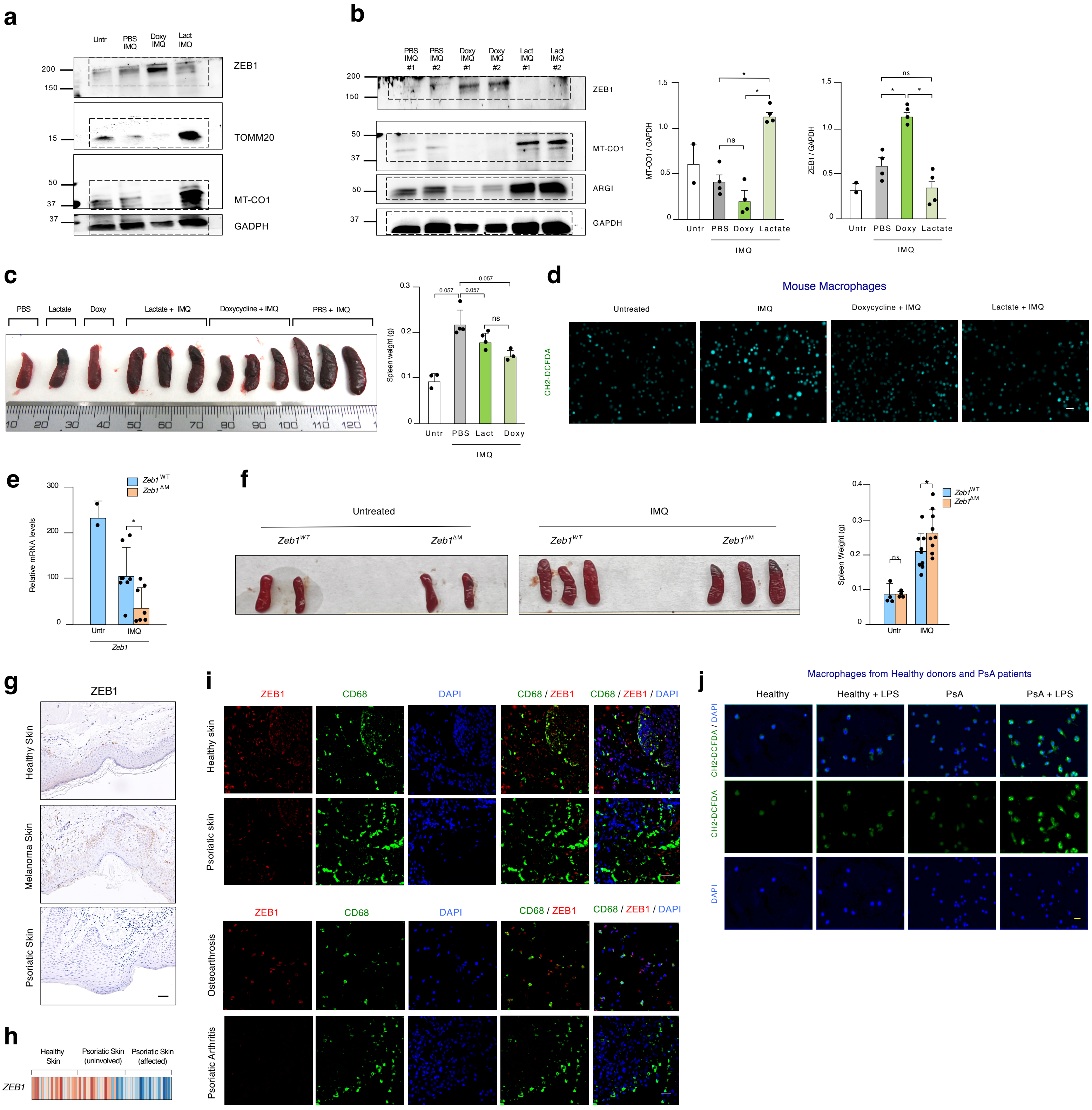

## SUPPLEMENTARY FIGURE S5

**Metformin requires ZEB1 expression in macrophages for its anti-inflammatory effects in psoriatic lesions. (A*)*** Uncropped blots for Fig. 5E. **(B)** As in Fig. 5A, cell lysates from *Zeb1*$^{WT}$ macrophages treated with imiquimod (IMQ), doxycycline + imiquimod, or lactate + imiquimod were blotted for MT-CO1 (PA1317-1, 1/400), ARGINASE-1 (V-20, 1/400) ZEB1 (H3, 1/200) and GAPDH (ab56783, 1/3000). *Left Panel:* Representative blots from three experiments are shown. *Right panel*: quantification of MT-CO1 and ZEB1 protein expression relative to GAPDH (n=4). **(C)** *Zeb1*$^{WT}$ mice either untreated (PBS), or treated with lactate, doxycycline, imiquimod, lactate + imiquimod, or doxycycline + imiquimod were assessed for spleen size and weight. *Left panel*: Representative pictures of spleens from at least three mice per condition. *Right panel*: Quantification of spleen weights (n=3,4,3,4 mice). **(D)** Immunocytochemistry for $CH_2$-DCFDA of peritoneal macrophages from *Zeb1*$^{WT}$ mice either untreated (PBS) or treated with imiquimod, doxycycline + imiquimod, or lactate + imiquimod. **(E)** *Zeb1* mRNA levels in the ears of *Zeb1*$^{WT}$ and *Zeb1*$^{\Delta M}$ mice either untreated or treated with imiquimod. Data are the average of two independent experiments each with two untreated mice and 3-4 imiquimod-treated mice per genotype and condition (n=2,7,7 mice). **(F)** Assessment of the spleens of untreated *Zeb1*$^{WT}$ and *Zeb1*$^{\Delta M}$ either untreated (PBS) or treated with imiquimod. *Left panel:* Representative pictures from three independent experiments, each with at least three mice per condition and genotype. *Right panel:* Quantification of spleens weight (n=4,4,9,9). **(G)** Immunohistochemistry staining for ZEB1 (AMAb90510; 1/100) in skin samples of healthy donors as well as melanoma and psoriatic patients. Pictures are representative pictures from at least three donors/patients. Scale bars: 20 µm. **(H)** Heatmap for ZEB1 expression in healthy and psoriatic affected and non-affected skin samples from the published array reference GSE14905 (4). **(I)** Additional staining combinations for Fig. 5I. Scale bar: 25 µm. **(J)** Human monocyte-derived macrophages from healthy donors or PsA patients, either untreated or treated with LPS, were stained for $CH_2$-DCFDA along with DAPI. Captions shown are representative of two healthy donors and one PsA patient. Scale bar: 25 µm. Graph bars in Figure represent mean values +/- SEM with two-tailed unpaired Mann-Whitney test. $p \leq 0.001$ (***), $p \leq 0.01$ (**) or $p \leq 0.05$ (*) levels, or non-significant (ns) for values of $p > 0.05$. Raw data along p values for statistical analyses are included in the Source Data file.

# Supplementary Figure S6

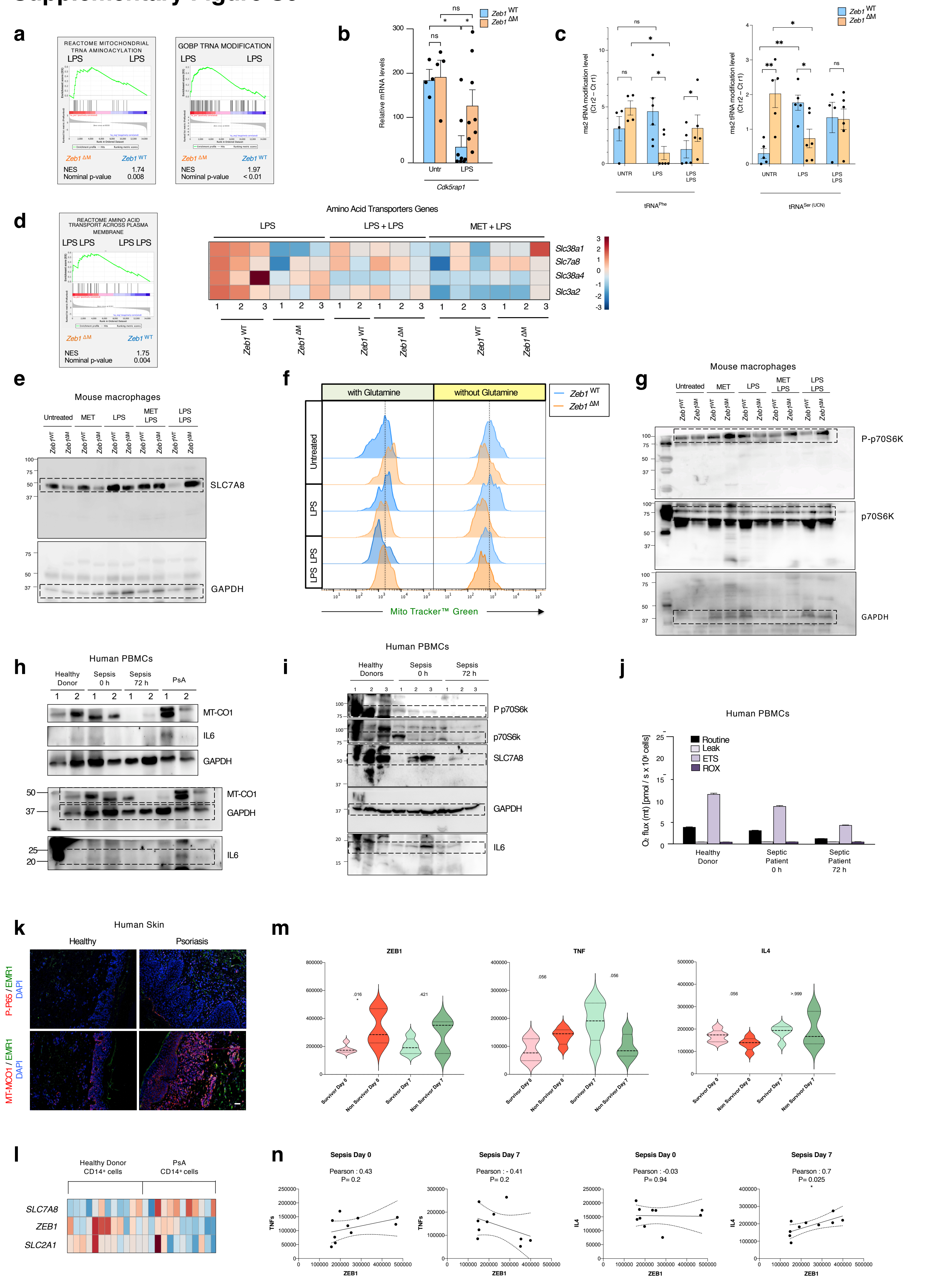

## SUPPLEMENTARY FIGURE S6

**ZEB1 inhibits mitochondrial protein translation by restricting amino acid transport. (A)** GSEA plot for mitochondrial tRNA aminoacylation and tRNA modification signatures comparing macrophages from *Zeb1*$^{WT}$ and *Zeb1*$^{\Delta M}$ mice treated with LPS. **(B)** *Cdk5rap1* mRNA levels in *Zeb1*$^{WT}$ and *Zeb1*$^{\Delta M}$ macrophages either untreated or treated with LPS (n=4,4,8,8). **(C)** tRNA$^{Phe}$ and tRNA$^{Ser(UCN)}$ ms2 modifications in macrophages isolated from *Zeb1*$^{WT}$ and *Zeb1*$^{\Delta M}$ mice either untreated or treated with LPS or LPS+LPS. Data represent the average of three independent experiments, with 1-2 mice per genotype and condition (n=5). **(D)** *Left Panel*: GSEA plot for amino acid transport signature comparing macrophages from *Zeb1*$^{WT}$ and *Zeb1*$^{\Delta M}$ mice treated with "LPS + LPS". *Right Panel*: Heatmap of glutamine-transporter genes in peritoneal macrophages from *Zeb1*$^{WT}$ and *Zeb1*$^{\Delta M}$ mice treated with LPS, LPS + LPS, or metformin + LPS. **(E)** Uncropped blots for Fig. 6C. **(F)** MTG staining in macrophages from *Zeb1*$^{WT}$ and *Zeb1*$^{\Delta M}$ mice either untreated or treated with LPS or "LPS + LPS" in the presence or absence of glutamine. The plot shown is representative of two mice per genotype and condition. **(G)** Uncropped blots for Fig. 6I. **(H)** Cell lysates from human PBMCs from healthy donors, septic patients at 0 h and 72 h, and PsA patients (n=2) were blotted for MT-CO1 (PA1317-1, 1/300), IL6 (MAB206, 1/400) and GAPDH (14C10, 1/2500). Uncropped blots are shown below. **(I)** Uncropped blots for Fig. 6J. **(J)** The mitochondrial respiratory activity of human PBMCs from healthy donors, septic patients at 0 h and 72 h after ICU admission, patients were measured by Oroboros $O_2$k high-resolution respirometry. Data are representative of three healthy donors, 4 septic patients at 0 h, and 3 septic patients at 72 h (n=3,4,3). **(K)** Immunofluorescence staining of skin samples from healthy donors and psoriatic patients for P-p65 (P-P6527.Ser 536, 1/100), EMR1 (BM8, 1/100) along with DAPI. Captions are representative of at least two independent experiments. Scale bar: 20 µm. **(L)** Heatmap of *SLC2A1, ZEB1*, and *SLC7A8* expression in peripheral blood CD14+ monocytes from healthy donors and PsA patients from published array GSE57383 (5). **(M)** Expression of *ZEB1*, *TNF*, and *IL4* in PBMCs of septic patients that either survive (survivors) or did not survive (non-survivors) at the time of diagnosis of sepsis (day 0) or seven days later (day 7) from GSE48080 (n=5 patients per group) (6). **(N)** As in (M), scatterplot of *ZEB1* between *TNF* or *IL4* at days 0 and 7. The Pearson correlation coefficient ($\rho$) and statistical significance (p value) are shown above the graphs. Graph bars in Figure represent mean values +/- SEM with two-tailed unpaired Mann-Whitney test. $p \le 0.001$ (***), $p \le 0.01$ (**) or $p \le 0.05$ (*) levels, or non-significant (ns) for values of $p > 0.05$. Raw data along p values for statistical analyses are included in the Source Data file.

# SUPPLEMENTARY TABLES

## Supplementary Table S1: Data of Healthy volunteers (n = 13) *

| Characteristics | Values |
|---|---|
| Age (years) | 33.4 (22-45) |
| Sex (male/female), n (%) | 3/10 (23.1/76.9) |

* Data are expressed as median (interquartile range) or absolute count (percentage)

**Supplementary Table S2: Basic clinical data of septic patients at the time of admission in the ICU (n = 20) ***

| Clinical characteristics | Values |
|---|---|
| Sepsis classification<br>Sepsis<br>Septic shock | <br>6 (30%)<br>14 (70%) |
| Sex<br>Male<br>Female | <br>10 (50%)<br>10 (50%) |
| Age (years) | 56 (29-85) |
| Comorbidities<br>Hypertension<br>Heart disease<br>Diabetes<br>Pneumopathy<br>Chronic kidney disease<br>Immunosuppression | <br>9 (45%)<br>6 (30%)<br>3 (15%)<br>1 (5%)<br>1 (5%)<br>0 |
| Site of infection<br>Urinary<br>Pulmonary<br>Biliary<br>Endovascular<br>Central Nervous System<br>Gastrointestinal<br>Gynecological<br>Cutaneous | <br>6 (30%)<br>5 (25%)<br>3 (15%)<br>2 (10%)<br>1 (5%)<br>1 (5%)<br>1 (5%)<br>1 (5%) |
| Microorganisme<br>*Klebsiella pneumoniae*<br>*Streptococcus pneumonie*<br>*Escherichia coli*<br>*Staphylococcus aureus*<br>*Enterococcus faecalis*<br>*Acinetobacter sp*<br>*Listeria monocytogenes*<br>*Streptococcus pyogenes*<br>*Chlamydia trachomatis*<br>No isolates | <br>4 (20%)<br>2 (10%)<br>2 (10%)<br>2 (10%)<br>1 (5%)<br>1 (5%)<br>1 (5%)<br>1 (5%)<br>1(5%)<br>1(5%) |
| Cause of hospital admission<br>Medical<br>Surgical | <br>18 (90%)<br>2 (10%) |
| CRP (mg/dL) | 24 (12-28) |
| Lactate (mg/mL) | 30 (16-38) |
| APACHE II score | 18 (15-23) |
| SOFA II score | 10 (9-11) |
| Vasopressors | 20 (100%) |
| Invasive mechanical ventilation | 3 (15%) |
| Renal replacement therapy | 1 (5%) |
| Hospital length of stay (days) | 11 (6-23) |
| ICU length of stay (days) | 3 (2-10) |
| 28-day mortality | 2 (10%) |

* Data are expressed as median (interquartile range) or absolute count (percentage)

Abbreviations: CRP: C-Reactive Protein. APACHE: Acute Physiology and Chronic Health Evaluation. SOFA: Sequential Organ Failure Assessment. ICU: Intensive Care Unit.

**Supplementary Table S3: Clinical data of PsA patients (n = 12) ***

| Clinical characteristics | Values (frequency, median, or %) |
|---|---|
| Age (years)* | 53.5 (13.5) |
| Sex (male/female), n (%) | 7/5(58.3/41.7) |
| Disease duration (years)* | 8.9 (7.8) |
| TJC* | 1.8 (2.3) |
| SJC* | 1.9 (1.8) |
| VAS pain* | 2.6 (8.4) |
| DAS28-ESR* | 3.27 (1.08) |
| DAS28-CRP* | 2.59 (0.78) |
| DAPSA* | 11.55 (7.48) |
| ESR (mm/1h) * | 29 (33) |
| CRP (mg/dL) * | 1.41 (1.43) |
| **Therapy taken** | |
| csDMARDs, n (%) | 2 (16.7) |
| BIOL, n (%) | 5 (41.7) |
| None n (%) | 5 (41.7) |

* Figures outside parenthesis are the median. Data inside the parenthesis are the standard deviations.

Abbreviations: BIOL: biological therapy; CRP: C-Reactive Protein; csDMARD: Disease-Modifying antirheumatic drug; DAPSA: Disease Activity in Psoriatic Arthritis; DAS: Disease Activity Score; ESR: erythrocyte sedimentation rate; SJC: Swollen Joint Count; TJC: tender Joint Count; VAS: Visual Analog Scale.

**Supplementary Table S4**: sgRNA and ssDNA oligonucleotides used in the generation of the *Zeb1*$^{fl/fl}$ mouse *

| sgRNA oligonucleotides | Sequence |
|---|---|
| sgRNA 5’ | 5’-TTACAGACACCTCTAACACAAGG-3’ |
| sgRNA 3’ | 3’-AGTACCAGCAAACCCTTTCTTGG-5’ |
| **sgRNA oligonucleotides** | **Oligos for cloning into the px330 vector** |
| sgRNA 5’ | Forward: CACCgttacagacacctctaacaca<br>Reverse: AAACtgtgttagaggtgtctgtaaC |
| sgRNA 3’ | Forward: CACCgagtaccagcaaaccctttct<br>Reverse: AAACagaaagggtttgctggtactC |
| **ssDNA oligonucleotides** | **Sequence** |
| ssDNA #1 | 5’- agctaagtcccttcaagtgcctggtcactgaggaaagctggggTTACAGACACCTCTAAC GCTAGCataacttcgtatagcatacattatacgaagttatACAAGGcttcctccccaaaagggagccgtaca gacatgaaaatatttatcaatcaaaggc - 3’ |
| ssDNA #2 | 3’- aaccaaaggttaacctaactcctaacaaaggagttggcacacgaAGTACCAGCAAACCCTGA ATTCataacttcgtataatgtatgctatacgaagttatTTCTTGGctttatggtgaatgggaacatggttgtttaat agtgatcataagcaaagaaga - 5’ |

* The code for colored bases is as follows: blue bases refer to the sgRNA sequence, red bases refer to the protospacer adjacent motif, bases in orange refer to the LoxP sequences while bases in green indicate the restriction enzyme target. Underlined bases correspond to the homology arms.

**Supplementary Table S5**: Primary and Secondary Antibodies

| Unconjugated Primary Antibodies | | |
|---|---|---|
| **Target protein** | **Source** | **Clone (Catalog Number)** |
| β-actin | Santa Cruz Biotechnology | C4 (sc-47778) |
| ADGRE1 (F4/80) / EMR1 | Santa Cruz Biotechnology | BM8 (sc-52664) |
| Arginase I | Santa Cruz Biotechnology | V-20 (sc-18354) |
| CD68 | Santa Cruz Biotechnology | KP1 (sc-20060) |
| GAPDH | Cell Signaling | 14C10 (2118L) |
| GAPDH | Proteintech | 1E6D9 (60004-1-Ig) |
| IL6 | ImmunoTools GmbH | IT1G2 (22450061) |
| IL6 | R&D Systems | 6708 (MAB206) |
| MT-CO1 | Booster Biological Technology | PA1317-1 |
| p62 (SQSTM1). | Santa Cruz Biotechnology | D-3 (sc-28359) |
| p70(S6K) | Proteintech | 14485-1-AP |
| Phosphorylated p65 (P-p65) | Santa Cruz Biotechnology | 27.Ser 536 (sc-136548) |
| Phospho-p70(S6K) (Thr389) | Proteintech | 28735-1-AP |
| SLC7A8 (LAT2) | ImmunoGlobe | (20180105) |
| SLC7A8 (LAT2) | Origene | OTI5A9 |
| TOMM20 | Abcam plc | ab56783 |
| ZEB1 | Santa Cruz Biotechnology | H-3 (sc-515797) |
| ZEB1 | Home-made (2) | N/A |
| ZEB1 | Sigma-Aldrich | AMAb90510 |
| ZEB1 | Sigma-Aldrich | HPA027524 |
| **Conjugated Primary Antibodies** | | |
| **Target protein** | **Source** | **Clone (Catalog Number)** |
| CD11b: anti-mouse CD11b PE-conjugated | ImmunoTools GmbH | M1/70.15 (22159114) |
| CD11c: Anti-CD11c Armenian Hamster Monoclonal Antibody (PE (Phycoerythrin)/Cy7®) | ImmunoTools GmbH | N418 |
| CD14: anti-human CD14 PerCP-conjugated | ImmunoTools GmbH | 18D11 (21620145) |
| CD45: anti-mouse CD45-conjugated PerCP/Cy5.5 | BioLegend | 30-F11 (103132) |
| F4/80: Alexa Fluor® 488 anti-mouse Ab. Rat IgG2a, κ | BioLegend | BM8 (123119) |
| F4/80: APC anti-mouse mAb. Rat IgG2a, κ | Biolegend | BM8 (123116) |
| GR-1: FITC- conjugated monoclonal antibody | ImmunoTools GmbH | RB6-8C5 |
| IL-6: anti-mouse IL6 PE-conjugated. Rat IgG1, κ | Biolegend | MP5-20F3 |
| Ly-6C: Ly-6C Monoclonal Antibody, PerCP-Cyanine5.5 | eBioscience™ | HK1.4 |
| **Conjugated protein** | **Source** | **Catalog Number** |
| Annexin V: APC conjugated | ImmunoTools GmbH | 31490016 |
| **Secondary Antibodies and Normal Sera** | | |
| Alexa Fluor® 488 AffiniPure Donkey Anti-Mouse IgG (H+L) | Jackson ImmunoResearch | 715-545-150 |
| Peroxidase-AffiniPure Donkey Anti-Mouse IgG (H+L) | Jackson ImmunoResearch | 715-035-151 |
| Rhodamine Red-X-AffiniPure Donkey Anti-Mouse IgG (H+L) | Jackson ImmunoResearch | 715-295-151 |
| Alexa Fluor 488-AffiniPure Donkey Anti-Rabbit IgG (H+L) | Jackson ImmunoResearch | 711-545-152 |
| Rhodamine Red™-X (RRX) AffiniPure Donkey Anti-Rabbit IgG (H+L) | Jackson ImmunoResearch | 711-295-152 |
| Normal Donkey Serum | Jackson ImmunoResearch | 017-000-121 |
| Mouse Gamma Globulin | Jackson ImmunoResearch | 015-000-002 |

**Supplementary Table S6**: DNA primers used in qRT-PCR

| Human Genes | Forward 5'- 3' | Reverse 3'- 5' | Ref |
|---|---|---|---|
| *ACTB* | CCCAGCACAATGAAGATCAA | GATCCACACGGAGTACTTG | 7 |
| *CDK5RAP1* | GGAGGAAGATCACGTCCAGA | ACTGAGCCCTTCCACTAGCA | 8 |
| *IL4* | AGCGAGTGTCCTTCTCATGG | CAGCCTCACAGAGCAGAAGA | 9 |
| *IL6* | CACAGACAGCCACTCACCTC | TTTTCTGCCAGTGCCTCTTT | 10 |
| *SLC2A1* | CTTTGTGGCCTTCTTTGAAGT | CCACACAGTTGCTCCACAT | 11 |
| *SLC7A8* | AACCTTCCCAGAGCCATCTT | GTGGACAGGGCAACAGAAAT | 12 |
| *SQSTM1* | GAGAGTGTGGCAGCTGCCCT | GGCAGCTTCCTTCAGCCC | 13 |
| *ZEB1* | GCCAATAAGCAAACGATTCTG | TTTGGCTGGATCACTTTCAAG | 14 |
| **Mouse Genes** | **Forward 5'- 3'** | **Reverse 3'- 5'** | **Ref** |
| *Actb* | GGCTGTATTCCCCTCCATCG | CCAGTTGGTAACAATGCCATGT | 15 |
| *Cdk5rap1* | GAAGCAGCCGTGTACTGGAT | ATGCCACATATGCTTCTCTTGA | 16 |
| *Il4* | TTGAACGAGGTCACAGGAGA | AAATATGCGAAGCACCTTGG | 17 |
| *Il6* | TGGTACTCCAGAAGACCAGAGG | AACGATGATGCACTTGCAGA | 18 |
| *Mt-Nd3* | ACAAGCTCTGCACGTCTACC | GCTCATGGTAGTGGAAGTAGAAGAG | 19 |
| *Slc2a1* | GTCCTGCTGCTATTGCTGTG | CCTCGGGTGTCTTGTCACTT | 20 |
| *Slc7a8* | AGCGAAACAACACCGCGAAGAA | TTTCCAGCACACCTTTCGGT | 21 |
| *Sqstm1* | CTTGTAGTTGCATCACGTAGAG | CAGCATATGCACATTCAAAACTCTC | 22 |
| *Zeb1* | ATTCCCCAAGTGGCATATACA | GAGCTAGTGTCTTGTCTTTCATCC | 23 |

**<u>Supplementary Table S7</u>**: Oligonucleotides for ChIP assays

| Promoter Region | Forward 5'- 3' | Reverse 5'- 3' | Reference |
|---|---|---|---|
| *SQSTM1* promoter ZEB1 binding site (-285 bp) (-369/-187 bp) | CCCCAACTGAGGATATTGCT | TCATTGCTGACCCCTCTCTT | This article |

**Supplementary Table S8**: Oligonucleotides for measuring $tRNA^{Phe}$, $tRNA^{SerUCN}$, $tRNA^{Trp}$, and $tRNA^{Tyr}$ modifications

| tRNA | Primers | Sequence | Reference |
|---|---|---|---|
| $tRNA^{Phe}$ | Forward | GCTTAATAACAAAGCAAAGCA | 24 |
| | Reverse R1 | TATCCATCTAAGCATTTTCA | 24 |
| | Reverse R2 | TGGGATACAATTATCCATCT | 24 |
| $tRNA^{Ser(UCN)}$ | Forward | CATATAGGATATGAGATTGGC | 24 |
| | Reverse R1 | AACCCCCTAAAATTGGTTTCA | 24 |
| | Reverse R2 | GAAGGAATCGAACCCCCTAA | 24 |

## SUPPLEMENTARY METHODS

### Research Ethics Statement

The research conducted in this study adhered to all pertinent ethical guidelines. Please refer to the sections below for detailed information on the ethical approvals concerning the use of human samples and mouse models.

### Human samples

The use of human samples in this study was approved by the local Clinical Experimentation Ethics Committee at Hospital Clínic (Barcelona, Spain) (approval references HCB/2017/0767, HCB/2019/1012, HCB/2020/0100), and all donors and patients provided their informed consent for the use of samples. Whole blood and buffy coats from healthy donors were obtained through the Catalan Bank of Blood and Tissues (Barcelona, Spain). Peripheral blood from septic patients or patients with psoriatic arthritis was obtained from the Medical Intensive Care Unit and the Department of Rheumatology at Hospital Clinic (Barcelona, Spain), respectively. As controls, we included 13 healthy volunteers, 10 females and 3 males, age range criteria: between 22 and 45 years old, with an average of 33 years old (see Supplementary Table S1). Septic patients, age selection criteria between 29 to 85 years old (see Supplementary Table S2), admitted to the Intensive Care Unit (Hospital Clinic of Barcelona, Spain) within the first 24 h of developing symptoms, were prospectively included and classified as sepsis or septic shock according to the criteria established by the United States' Society of Critical Care Medicine and the European Society of Intensive Care Medicine third consensus conference definitions (2016, Sepsis-3) (25). Patients who met any of the following criteria were excluded from the study: pregnancy, patients with active onco-hematological disease, infection with the human immunodeficiency and/or hepatitis C viruses, under previous treatment with corticosteroids, inclusion in a clinical trial in the previous month, patients with life expectancy less than 6 months, with “Do Not Resuscitate” establishment orders or futile care prior to enrollment, and patients with a septic shock derived from an unresolved surgical problem. The severity of illness and organ dysfunction were assessed with the Acute Physiologic and Chronic Health Evaluation (APACHE)-II (26) and the Sequential Organ Failure Assessment (SOFA) scores (27). Psoriatic arthritis (PsA) patients, aged between 26 and 72 years old (see Supplementary Table S3) under clinical control at the Arthritis Unit, Department of Rheumatology (Hospital Clinic, Barcelona, Spain) were diagnosed according to the Classification Criteria for Psoriatic Arthritis (CASPAR) criteria (28). Patients were in active disease according to Disease Activity Score using 28 joint counts (DAS28) and Disease Activity Index for Psoriatic Arthritis (DAPSA) scores (29). Peripheral blood and synovial membrane from these patients were matched with blood samples from healthy controls and synovial membrane from osteoarthritis patients according to classification criteria. Skin samples of psoriatic and melanoma patients were obtained from the Dept. of Pathology at Hospital Clínico San Carlos (Madrid, Spain). Peripheral blood mononuclear cells (PBMCs) from healthy controls, septic patients or PsA patients were isolated by differential density centrifugation over Ficoll-Paque Premium 1.073 (GE Healthcare Life Science). Wherever indicated, PBMCs were used to generate macrophages as described elsewhere (30). PBMCs were plated, and non-adherent cells were removed by washing with PBS, followed by a 6-day treatment with 20 ng/ml of human recombinant CSF1 (ImmunoTools GmbH, Friesoythe, Germany). In the “Untreated” condition, human monocyte-derived macrophages (MDM) were treated with PBS for 27-39 h. In the "LPS" condition, macrophages were initially treated with PBS for 24-36

h, followed by treatment with 100 ng/ml of LPS for 2-3 h. In the metformin (MET) protocol, macrophages were treated with 2 mM hydrochloride metformin for 24-36 h, followed by exposure to PBS for an additional 2-3 h. The metformin + LPS condition involved the pre-treatment of macrophages with 2 mM hydrochloride metformin or 24-36 h, followed by treatment with 100 ng/ml of LPS for 2-3 h. Finally, in the "LPS + LPS" protocol, macrophages were pre-treated with 100 ng/ml of LPS for 24-36 h, followed by an additional dose of 100 ng/ml of LPS for an additional 2-3 h. For all treatment groups, macrophages were collected 2-3 h after the last stimuli for further analysis.

**Generation of *Zeb1*$^{fl/fl}$ and *Zeb1*$^{\Delta M}$ mice**

The conditional *Zeb1* flox allele mouse (*Zeb1*$^{fl/fl}$, herein referred as *Zeb1*$^{WT}$) (mouse model reference: B6.B6CBA-*Zeb1*em1/cnbbm) was generated in the joint National Biotechnology Center (CSIC-CNB) / Severo Ochoa's Molecular Biology Center (CSIC-CBMSO) Transgenesis Unit at the Spanish National Research Council (CSIC) and Autonomous University of Madrid (Madrid, Spain). Two gRNAs (sgRNA5´and sgRNA 3´) were designed using the Breaking-Cas online platform (Oliveros et al., 2016) (https://bioinfogp.cnb.csic.es/tools/breakingcas/) (see Supplementary Table S4) to elicit double-strand breaks (DSBs) flanking exon 6 in the *Zeb1* gene. Two oligonucleotides for each targeting site (Supplementary Table S4) were annealed and ligated into the pX330-U6-Chimeric_BB-CBh-hSpCas9 vector (Addgene plasmid # 42230; http://n2t.net/addgene:42230; RRID: Addgene_42230) (31), which was a gift from F Zhang (Massachusetts Institute of Technology, Cambridge, MA, USA). T7 promoter sequence was then added to the sgRNA template for subsequent amplification by PCR and in vitro transcription (IVT) as previously described (32). In addition, two ssDNA oligos (see Supplementary Table S4), that contain the corresponding LoxP site and a restriction enzyme flanked by two 60 bp homology arms, which correspond to the sequence surrounding each Cas9 cut site, were designed and purchased from Sigma-Aldrich (Merck KGaA, Darmstadt, Germany). A mixture of in vitro transcribed RNA Cas9 (Sigma-Aldrich, Merck KGaA), two sgRNAs, and two ssDNAs (ssDNA 5' + ssDNA 3') was then injected into the cytoplasm of B6CBAF2 zygotes, using standard procedures (33). The concentrations of RNAs injected were as follows: 100 ng/µl for Cas9, 50 ng/µl for each sgRNA 5'- and sgRNA 3'-, and 100 ng/µl for each ssDNAs. Zygotes that survived the injections were transferred into the oviducts of pseudopregnant foster mothers for development to term. The progeny was then crossed five times with wild type C57BL6/Jcrl mice to generate the *Zeb1*$^{fl/+}$ mice (mice expressing one floxed allele and one wild type allele). The presence of correct LoxP sequences was analyzed by DNA sequencing (Supplementary Table S4). The *Zeb1*$^{fl/fl}$ (*Zeb1*$^{WT}$) mouse was then crossed with a mouse carrying the Cre recombinase selectively in myeloid cells under the control of the endogenous lysozyme 2 (*Lyz2*, also referred as *LysM*) promoter/enhancer (official name: B6.129P2-*Lyz2*$^{tm1(cre)Ifo}$/J), (34) (The Jackson Labs, Bar Harbor, ME, USA), to generate the myeloid conditional *Zeb1* knockout (*Zeb1*$^{fl/fl}$/*LysM*$^{Cre}$, referred in the manuscript as *Zeb1*$^{\Delta M}$) mice. The floxed and Cre sequences in experimental mice were genotyped by PCR amplification of genomic DNA extracted from tail samples. The mice were housed in a temperature-controlled barrier room maintained at 21-22°C with a 12-hour light/dark cycle. They were provided with standard rodent chow (RM1-P, SDS, Dietex, Argenteuil, France) and had access to water ad libitum. All mice were euthanized by cervical dislocation.

**Experimental mouse model of endotoxin septic shock**

Eight-to-ten weeks old female *Zeb1*$^{WT}$ and *Zeb1*$^{\Delta M}$ mice in Figure 1A and

throughout the paper were subjected to a model of sterile septic shock with lipopolysaccharide (LPS) (Escherichia coli 055:B5, Sigma-Aldrich). Mice were divided into separate cohorts, one of the cohorts was subjected to LPS-induced lethal endotoxemia (systemic acute inflammation, referred throughout the paper to as “LPS”), whereas the other cohort was subjected to LPS-induced immunosuppression (tolerance, referred throughout the paper to as LPS + LPS) (Supplementary Fig. S1A). In the cohort undergoing the LPS protocol, mice were injected intraperitoneally (i.p.) with 250 μl of vehicle (PBS) followed by i.p. injection of a single lethal dose of LPS (25 mg/kg) 24 h later. In the cohort subjected to the "LPS + LPS" protocol, mice received an intraperitoneal (i.p.) injection of a low sublethal dose of LPS (5 mg/kg), followed by an i.p. injection of a high lethal dose of LPS (25 mg/kg) 24 h later. In the mice subjected to metformin alone (MET) group, mice were injected i.p. with metformin hydrochloride (150 mg/kg in PBS), followed by an i.p. injection of PBS (250 μl) 24 h later. In mice subjected to the metformin + LPS (MET + LPS) protocol, mice were injected i.p with hydrochloride metformin (150 mg/kg) followed by injection with a high lethal dose LPS (25 mg/kg) 24 h later. In the experiments shown in Figures 1A and 1B, mice were closely monitored every 2 h during the initial 12 h after the last LPS injection and subsequently every 4 h, excluding nighttime. This monitoring involved tracking movement, respiratory rate, body temperature and weight, as well as observing for any signs of distress. In accordance with the ethical guidelines in the approved protocol by the University of Barcelona Animal Experimental Committee (reference 396/18), mice must be euthanized when they reached the humane endpoints criteria (as approved in the approved protocol), namely lack of movement, respiratory distress, body temperature below 30ºC, or weight loss exceeding 20%. This resulted in the euthanasia of one *Zeb1*$^{\Delta M}$ mouse in the LPS + LPS condition before the end of the protocol. The number of mice included in the septic shock experiments is in line with the number of mice included in the literature (35,36). A total of 15 *Zeb1*$^{WT}$ and 12 *Zeb1*$^{\Delta M}$ mice were subjected to the LPS protocol and followed for 30 h; only 5 *Zeb1*$^{WT}$ and *Zeb1*$^{\Delta M}$ mice survived until the end of the protocol. A total of 14 *Zeb1*$^{WT}$ and *Zeb1*$^{WT}$ mice were subjected to the LPS + LPS protocol and followed for 90 h with 11 *Zeb1*$^{WT}$ and 4 *Zeb1*$^{WT}$ mice surviving until the end. A *Zeb1*$^{\Delta M}$ mouse in the LPS + LPS protocol had to be euthanized after only 45 h due to ethical considerations. Apart from the experiments shown in Figures 1A and 1B, in all other experiments of the study, mice subjected to PBS, LPS, metformin, metformin + LPS, or LPS + LPS were euthanized 2-3 h after the last injection, and their peritoneal macrophages or peripheral blood samples were collected for further analyses.

**Imiquimod-induced mouse model of chronic inflammation**

The imiquimod (IMQ)-induced mouse model of psoriasiform skin inflammation was set as described elsewhere (37,38). Mice were subjected to the following pretreatments 24 h before the administration of imiquimod in both ears (see Fig. 5A): intraperitoneal injection of 250 μl of PBS as control (referred to in the text and figures as Untreated), metformin (150 μg/g total weight of metformin hydrochloride, Sigma-Aldrich), L-lactate (2 μg/g total weight, Sigma-Aldrich, in PBS) or Doxycycline Hydrochloride (60 μg/g of total weight, Fisher Scientific). These pre-treatments were continued for 7 days. For each mouse were then one of the ears was left untreated while the other was treated topically daily for 6 days with 35 mg of imiquimod cream (Aldara 5%, MEDA Pharma S.L., Madrid, Spain). After euthanasia, peritoneal cells were collected, and macrophages were enriched by adherence to tissue culture plates as described above. Ear skin samples were first fixed overnight in 3.7-4% formaldehyde solution followed by

incubation for 24 h in 30% ethanol. Samples were then embedded in paraffin and processed for subsequent histological analysis.

**Isolation and culture of mouse macrophages**

Peritoneal macrophages were isolated from 6- to 8-week-old *Zeb1*$^{WT}$ and *Zeb1*$^{\Delta M}$ mice as per standard protocols (39). Briefly, mice were euthanized, and the peritoneal cavity was washed twice with 6 ml of ice-cold PBS supplemented with 3% FBS. Cells from the peritoneal lavage were centrifuged at 400 × g for 10 min at 4ºC, and erythrocytes were osmotically lysed with red blood cell lysis buffer (Sigma-Aldrich), followed by a wash with PBS and resuspension in PBS or complete medium. Peritoneal macrophages were sorted from peritoneal cells using flow cytometry based on their CD45$^+$, CD11b$^+$, and F4/80$^+$ expression profile. Wherever indicated, peritoneal macrophages were enriched by plating 5-10 x $10^5$ peritoneal cells on 6- or 12-well plates (Thermo Scientific, Waltham, MA, USA) for 30 minutes, followed by two washes with PBS to remove non-attached cells. In the *in vitro* experiments of acute inflammation and immunosuppression, macrophages were subjected to the following protocols (see scheme in Supplementary Fig. S1A and Fig. 3A). As for human MDM, mouse peritoneal macrophages were left untreated (PBS), treated with LPS, metformin + LPS, or LPS + LPS. For all treatment groups, macrophages were collected 2-3 h after the last stimuli for further analysis of cytokine protein levels as well as lactate, glucose, and glutamine levels. In the analysis of mRNA levels by qRT-PCR and protein levels by Western blot in mouse peritoneal macrophages, peritoneal cells were incubated in 5 ml round bottom tubes (SPL Life Sciences, Pocheon-si, Gyeonggi-do, South Korea) with “complete DMEM” [containing Dulbecco's modified Eagle medium (DMEM) (Lonza, Basel, Switzerland), 10% FBS (Sigma-Aldrich), and 1% penicillin-streptomycin (Pen/Strep) (Lonza)] and the corresponding stimulus for the indicated time in each treatment group. As per standard protocols (40), peritoneal macrophages were enriched by plating 5-10 x $10^5$ peritoneal cells onto 6- or 12-well plates (Thermo Scientific, Waltham, MA, USA) for 30 minutes. Cells were subsequently washed twice with PBS to remove non-attached cells and subsequently processed for protein or RNA analyses.

**Cell flow cytometry analysis and sorting**

Cells were first blocked for Fc receptors with mouse gamma globulin (Jackson ImmunoResearch Europe, Ely, United Kingdom) and then incubated with the corresponding fluorochrome-labeled antibodies in PBS with 2% FBS for 45 min at 4ºC (See Supplementary Table S5). For IL-6 intracellular staining, peritoneal cells were fixed and permeabilized with eBioscience™ Foxp3 / Transcription Factor Staining Buffer Set (00-5523-00, Thermo Fisher Scientific), and stained with IL-6 antibody (MP5-20F3, 1/100) for 2 h at 4ºC. The expression of cell surface proteins was assessed using a BD FACSCanto™ II analyzer (BD Biosciences, San Jose, CA, USA). In select experiments, cells were sorted for specific subpopulations using a FACS Aria™ II cell sorter (BD Biosciences) for subsequent experimentation. In specific experiments, human PBMCs or mouse peritoneal cells were stained for 30 min with CD14 (18D11, 1/100) or F4/80 (BM8, 1/200), respectively along with either 50 nM Mitotracker™ Green FM (MTG) (Thermo Fisher Scientific) and 100 nM tetramethylrhodamine methyl ester perchlorate (TMRM) (Thermo Fisher Scientific) in complete DMEM medium, or, whenever indicated, in DMEM high glucose without glutamine (Gibco). For ROS determination, cells were stained with 5 μM 2',7'-dichlorodihydrofluorescein diacetate ($CH_2DCFDA$) (Thermo Fisher Scientific) in PBS at 37°C and 5% CO2 for 30 min. FACS data were collected using FACSDiva Software Version 6.1. The acquired data were then analyzed using

FlowJo for Mac, version 10.8.1 (FlowJo®, Ashland, OR, USA).

### Protein expression analysis by Western blot

Mouse peritoneal cells or human PBMCs were washed with ice-cold PBS and then resuspended directly in RIPA lysis buffer (150 mM NaCl, 50 mM Tris pH 8, 1% NP-40, 0.5% SDS, 2 mM EDTA) containing protease inhibitors as described elsewhere (41). The lysates were clarified by centrifugation multiple times, and the protein concentration was determined using the Lowry assay. Equal amounts of protein in the lysates were subsequently boiled and loaded onto Novex™ WedgeWell™ 4-20% Tris-Glycine polyacrylamide gels (Invitrogen) for electrophoresis and then transferred to a PVDF membrane (Immobilon-P, Millipore, Bedford, MA, USA). Membranes were blocked with 5% non-fat milk and blotted with the following primary antibodies: β-ACTIN (clone C4, 1/500 dilution), GAPDH (either 2118L, 1/3,000 or 1E6D9, 1/15000), IL6 (either 6708, 1/400 or IT1G2, 1/200), LAT2/SLC7A8 (either OTI5A9, 1/500 or 20180105, 1/1000), MT-MCO1 (PA1317, 1/200), p62 (D-3 1/400), phosphorylated p65 (27.Ser 536, 1/800), TOMM20 (ab56783, 1/200), p70S6K (14485-1-AP, 1/2000), Phospho-p70S6K (Thr389) (28735-1-AP, 1/1000), ArgI (V-20, 1/400), and ZEB1 (either H-3, 1/200 or Home-made, Guo et al., 2021, dilution: 1/1000) antibodies for overnight at 4ºC. The source of the antibodies is detailed in Supplementary Table S5. After washing with TBS-T (137 mM NaCl, 20 mM Tris, 0.1% Tween-20, pH 7.6), membranes were incubated for 2 h at room temperature with the corresponding HRP-conjugated secondary antibody, either anti-rabbit (1:10,000) or anti-mouse (1:5,000). The chemiluminescent reaction was detected with Clarity™ ECL Western Blotting Substrate (Bio-Rad, Hercules, CA, USA). Uncropped gel images are shown in Data Source and the corresponding Supplementary Figure, with the target protein enclosed by a dashed box.

### RNA extraction and quantitative real-time PCR

Total RNA was extracted with TRIzol® (Life Technologies, Thermo Fisher Scientific) and reverse-transcribed with oligo-dT using High-Capacity cDNA Reverse Transcription Kit (Life Technologies). mRNA levels were then determined by quantitative real-time PCR (qRT–PCR) at 60°C using GoTaq® qPCR Master Sybr Green Mix (Promega Corp., Madison, WI, USA) in a LightCycler ® 96 real-time PCR apparatus (Roche, Rotkreuz, Switzerland). Results were analyzed using LightCycler® 96 Software Version 1.1 (Roche) by ΔΔCt method and normalizing values with respect to *Actb* (mouse) or *ACTB* (human) as the reference gene. The qRT-PCR data shown represent the average results obtained from a minimum of three mice of each genotype, with each sample analyzed in triplicate. DNA primers used in qRT–PCR were purchased from Sigma-Aldrich, and their sequences are described in Supplementary Table S6. Throughout the study, the nomenclature for human and mouse genes used adheres to the HGNC (HUGO Gene Nomenclature Committee, http://www.genenames.org/), and MGI (Mouse Genome Informatics, http://www.informatics.jax.org/) nomenclatures, respectively.

### RNA sequencing and data analysis

Peritoneal macrophages ($CD45^+$, $CD11b^+$, $F4/80^+$) from 6- to 8-week-old *Zeb1*$^{WT}$ and *Zeb1*$^{\Delta M}$ female mice—9 mice for each genotype and condition (LPS, LPS+LPS, MET+LPS)—were isolated and their RNA was extracted using TRIzol® (Life Technologies, Thermo Fisher Scientific). RNA was quantified and its quality (RNA integrity numbers ≥ 8.5) was assessed on an Agilent TS-4200 TapeStation (Agilent Technologies, Santa Clara, CA, USA). Part of the RNA samples was reverse-transcribed as described above to examine *Zeb1* expression. To obtain at least 10 ng of RNA required for the preparation of libraries in triplicate, macrophages from three mice samples

from each genotype and condition were pooled. The libraries from the mouse total RNA were prepared using the TruSeq® Stranded mRNA LT Sample Prep Kit (Rev.E, October 2013) (Illumina Inc., San Diego, CA, USA) according to the manufacturer's protocol. Briefly, 0.5 µg of total RNA was used for poly-A-based mRNA enrichment with oligo-dT magnetic beads. The mRNA was fragmented (fragment size: 80-250 nt, with the major peak at 130 nt) and the first-strand cDNA synthesis was conducted by random hexamers and reverse transcriptase. The second-strand cDNA synthesis was performed in the presence of dUTP instead of dTTP, to achieve strand specificity. The blunt-ended double-stranded cDNA was 3' adenylated and Illumina indexed adapters were ligated. The ligation product was then enriched with 15 PCR cycles, and the final library was validated on an Agilent 2100 Bioanalyzer with the DNA 7500 assay. Libraries were sequenced on HiSeq 2000 (Illumina, Inc) in a paired-end mode with a read length of 2 x 76 bp using TruSeq SBS Kit v3-HS. Over 30 million paired-end reads were generated for each sample in a fraction of a sequencing flow cell lane, following the manufacturer's protocol. Image analysis, base calling, and quality scoring of the run were processed using the manufacturer's software Real Time Analysis (RTA 1.13.48) and followed by the generation of FASTQ sequence files by Illumina's CASAVA. Reads were mapped against the mouse reference genome (GRCm38) with STAR (42) using the ENCODE parameters for long RNA. Gene quantifications were performed with RSEM (43) using default options and mouse GENCODE annotation version 22. Normalization and differential expression analyses were done with DESeq2 (44) using default options. Functional enrichment analysis of the differentially expressed genes was performed using the g:profiler platform (https://biit.cs.ut.ee/gprofiler/; 45), and gene set enrichment analysis was performed with the Preranked GSEA function (GSEA v4.3.2), (46,47) using the DESeq2 Wald test as the ranking statistic. Heatmap with the regularized log transformed expression of the 500 most variable genes was performed with the 'Pheatmap' R package and the option scale="row". Other heatmaps in the article were performed using the MetaboAnalystR 3.0 platform. From selected GO terms, we generated a cnetplot network hub representation using Cytoscape 3.9.1 including the highly statistically significant genes as described elsewhere (48,49). The RNA-seq data were submitted to the GEO database and assigned accession number GSE207328.

## Chromatin immunoprecipitation assays

Chromatin immunoprecipitation (ChIP) assays were performed using EpiQuick ChIP kit (Epigentek, Farmingdale, NY, USA) as per the manufacturer's instructions. Briefly, 1.5 x $10^7$ human monocyte-derived macrophages were incubated for 10 min with 1% formaldehyde solution (Electron Microscopy Sciences, Hatfield, PA, USA) at room temperature followed by incubation with 1.25 mM glycine. Lysates were sonicated as described elsewhere (8). Four µg anti-ZEB1 (clone H3) and its corresponding control mouse IgG (Jackson ImmunoResearch) were used. Identification of DNA binding sequences for ZEB1 and design of primers for qRT-PCR amplification were conducted using MacVector for Mac 18.6 software (MacVector Inc, Apex, NC, USA). DNA fragments were quantified by qRT-PCR as detailed above using the primers detailed in Supplementary Table S7. In all qRT-PCRs, values shown represent relative binding in relation to input and are the average of at least three independent ChIP experiments, each one performed in triplicate.

## Quantification of tRNA modifications

$tRNA^{Phe}$ and $tRNA^{Ser(UCN)}$ modifications in *Zeb1*$^{WT}$ and *Zeb1*$^{\Delta M}$ macrophages untreated or treated with LPS or LPS + LPS were assessed as described in

(24,50). The primers used are detailed in Supplementary Table S8.

**Determination of lactate levels**

Five x $10^5$ peritoneal macrophages isolated from LPS- or imiquimod-treated mice were cultured onto tissue culture-treated 12-well plates and incubated for 30 min in complete DMEM. Next, cells were washed twice with ice-cold PBS, and the adherent cells were subsequently cultured in 1 ml of DMEM supplemented with 2% FBS for 24 h. The supernatant was collected, and the levels of L-lactate were measured using a commercial kit (Amplite™ Colorimetric L-Lactate Assay Kit, AAT Bioquest, Sunnyvale, CA, USA) according to the manufacturer's instructions.

**In vivo assessment of reactive oxygen species (ROS)**

ROS production was assessed *in vivo* in female and male mice undergoing LPS and LPS+LPS protocols using the chemiluminescence probe L-012 (FUJIFILM Wako Chemicals USA, Corp., Richmond, VA, USA). Mice were i.p. injected with L-012 at a dose of 20 mg/kg in PBS. They were then anaesthetized with isoflurane, and their abdominal skin was shaved. The bioluminescence signal was captured using an IVIS® Lumina III In Vivo Imaging System (Revvity, Inc., formerly PerkinElmer, Waltham, MA, USA) and quantified using the Living Image® (Revvity, Inc) software.

**Determination of IL6 by enzyme-linked immunosorbent assay (ELISA)**

Mouse IL6 serum levels were determined using a commercial kit as per the manufacturer's instructions (ImmunoTools GmbH, Friesoythe, Germany). Briefly, whole blood extracted from the mandibular vein was spun down for 10 min at 3,000 x g, diluted 1:3 in PBS, and evaluated for absorbance at 450 nm wavelength using a Modulus II GloMax® Multi Detection System microplate reader (Promega, Madison, WI, USA). IL6 concentration was calculated against a reference standard curve included in the commercial kit. Data were analyzed with GainData® Arigo's ELISA calculator.

**Determination of cytokines by bead-based multiplex array**

Peritoneal macrophages were incubated with plain DMEM (without FBS) in the presence or absence of metformin hydrochloride (Sigma-Aldrich, Merck KGaA), LPS (Sigma-Aldrich, Merck KGaA), or PBS for 4 h. The supernatant was collected and centrifuged at 2,500 x g for 10 min at 4ºC to remove any remaining cell. The levels of a panel of 13 cytokines and chemokines (G-CSF, IFNγ, IL1β, IL2, IL4, IL6, IL12p70, IL13, IL17A, IL23p19, MCP-1, TNFα) were measured by FACS using a bead-based multiplex array (RayPlex™ Mouse Inflammation Array 1, FAM-INF-1-96, RayBiotech Life, Peachtree Corners, GA, USA) as per manufacturer's instructions. Cytokine levels in macrophage supernatants were assessed in a BD LSRFortessa™ Cell Analyzer (BD Biosciences, San Jose, CA, USA), quantified by comparing the fluorescence signal to a standard curve generated from purified protein standards at known concentrations, and concentration was corrected for protein levels in macrophage cultures assessed by a Lowry assay. Data were analyzed with GainData® Arigo's ELISA calculator.

**Determination of intracellular levels of glutamine/glutamate and Branched-Chain Amino Acids**

Approximately, 5 x $10^5$ peritoneal cells were incubated in a 96-well plate for 30 min, washed twice with PBS to select macrophages and incubated in complete DMEM, with the corresponding stimulus (PBS, metformin, LPS) for 36h. Then, the DMEM medium was replaced by complete DMEM including the respective stimulus (PBS, metformin, LPS) for 3 h. Macrophages were lysed using the buffer provided in the commercial kit, and intracellular levels of glutamine and glutamate, as well as of

branched-chain amino acids (BCAAs) were measured using commercial bioluminescent kits (Glutamine/Glutamate-Glo™ and BCAA-Glo™ assays, respectively, Promega Corp., Madison, WI, USA) following the respective manufacturer's recommendations.

**Assessment of macrophage glutamine uptake by liquid chromatography–mass spectrometry**

The supernatant of 5 x $10^5$ peritoneal macrophages treated with the indicated stimuli was collected 3 h after the last stimuli and diluted in 0.1% formic acid in water. Glutamine uptake was quantified by liquid chromatography-tandem mass spectrometry (LC-MS). Amino Acid Standard solution (AAS18, Sigma-Aldrich, Merck KGaA), containing 2.5 µmoL/mL of L-glutamic was used to correctly identify the levels of glutamine in the samples. The LC was run in an Eksigent MicroLC 200 Systems (AB Sciex Pte. Ltd., Singapore), with Avantor® ACE® AQ column (Hichrom Ltd., Reading, UK), 0.5 x 150 mm; flow rate 20 µL/min. This LC system was coupled to a 5500 QTRAP mass spectrometer (AB Sciex Pte. Ltd.) equipped with an electrospray ionization source. Data were acquired in MRM3, and the peaks were integrated using MultiQuant™ software v3.0.2 (AB Sciex Pte. Ltd.). The processed data were manually reviewed for quality and compared to confirm glutamine identity.

**Assessment of macrophage glucose uptake**

The supernatant of 5 x $10^5$ peritoneal macrophages cultured in complete DMEM and treated with the indicated stimuli was used to determine glucose levels using the Glucose Hexokinase method on an Atellica® CH 930 Analyzer (Siemens Healthineers, Erlangen, Germany).

**Relative mouse mtDNA copy number quantification**

Peritoneal cells were cultured with the indicated stimuli in 5 ml Round-Bottom Tubes (SPL Life Sciences) in DMEM high glucose without glutamine (Gibco) supplemented with 10% FBS, 1% penicillin-streptomycin (Lonza), 1 mM sodium pyruvate (Lonza), and either with or without 4 mM L-glutamine (Lonza). Peritoneal macrophages were subsequently enriched by plating 1 x $10^6$ cells on 6-well plates and washed twice with ice-cold PBS for 30 min. Total DNA was then extracted using the Wizard® Genomic DNA Purification Kit (A1120, Promega) as per manufacturer's instructions. The relative mtDNA copy number of peritoneal macrophages was quantified by using the Relative Mouse Mitochondrial DNA Copy Number Quantification qPCR Assay Kit (8938, ScienCell, Carlsbad, CA, USA) following the provided manufacturer's instructions in a LightCycler® 96 real-time PCR apparatus (Roche). Results were analyzed using LightCycler®96 Software Version 1.1 (Roche) by the ΔΔCt method and normalizing mtDNA values with respect to 100 bp-long region on mouse chromosome 10.

**Determination of ATP**

Five x $10^4$ peritoneal macrophages from *Zeb1*$^{WT}$ and *Zeb1*$^{\Delta M}$ mice were cultured in duplicate on tissue culture-treated 96-well plates for 30 min in complete DMEM. Cells were washed twice with ice-cold PBS and adherent cells were cultured in 200 µl of complete DMEM and either untreated, or treated with LPS, MET+LPS, or LPS+LPS. ATP levels in cells measured using a commercial kit (StayBrite™ Highly Stable ATP Bioluminescence Assay Kit) following manufacturers' instructions. ATP concentration in each well was corrected for protein levels using the Lowry assay.

**Cell immunofluorescence to assess mitochondrial content and membrane potential, ROS production, and mitophagy**

For immunofluorescence staining of human monocyte-derived macrophages and mouse peritoneal macrophages, cells were pelleted and plated on 24-well plates before being assessed for mitochondrial content and membrane

potential, ROS production, and mitophagy. To assess mitochondrial content and membrane potential and ROS production, cells were incubated with either 100 nM MTG (in complete DMEM, to assess mitochondrial content), 50 nM TMRM (in complete DMEM, to assess mitochondrial membrane potential), 5 µM $CH_2$-DCFDA (in PBS, to assess ROS production) for 30 min at 37ºC,, respectively. To asses mytophagy, we use the Mytophagy Detection kit (MD01-10, Dojindo Laboratories, Kumamoto, Japan). Briefly, 5 x $10^5$ peritoneal cells were plated in a µ-Slide 8 Well (80826, ibidi GmbH) incubated with 100 nM Mtphagy Dye$^{TM}$ (in DMEM) 30 min before the last LPS treatment and 1 µM Lyso Dye$^{TM}$ (in DMEM), 2.5 h after the last LPS treatment for 30 min at 37°C and 5% $CO_2$ as per manufacturers' instructions. For antibody cell staining, cells were fixed with 4% paraformaldehyde (Electron Microscopy Sciences, Hatfield, PA) for 20 min, and permeabilized with 0.25% Triton X-100 for 30 min. After washing three times with ice-cold PBS, cells were incubated with (see Supplementary Table S5 for their source): MT-CO1 (PA1317-1, 1/100), followed by incubation with Rhodamine X donkey anti-mouse (1/100). Cells were then counterstained with ProLong$^{TM}$ Gold Antifade Reagent with DAPI (Thermo Fisher). Staining was evaluated in a Nikon Eclipse E600 microscope (Nikon, Tokyo, Japan) and images were processed using ImageJ software (https://imagej.nih.gov/) (NIH, Bethesda, MD, USA).

**Tissue eosin and hematoxylin staining, and immunohistochemistry**

Glass slides containing formalin-fixed, paraffin-embedded human and mouse tissue samples were deparaffinized and rehydrated before being subjected for 5 min to antigen retrieval with 10 mM sodium citrate pH 6.0. In Fig. 4B and 4G, slides were only counterstained with hematoxylin (Sigma-Aldrich Merck KGaA) and eosin (Sigma-Aldrich, Merck KGaA) for 5 min. In immunohistochemistry staining, slides were blocked for endogenous peroxidase with 3% hydrogen peroxide in methanol for 15 min. Next, slides were incubated with a non-specific binding blocking solution [5% donkey normal serum plus 4% bovine serum albumin (BSA) in phosphate-buffered saline (PBS) containing 0.5% TWEEN-20® (Sigma-Aldrich, Merck KGaA)] followed by subsequent incubation with ZEB1 antibody (AMAb90510, 1:50 dilution) for overnight at 4°C and HRP-conjugated goat anti-rabbit IgG (1:100) for 1 h at 37°C. The immunohistochemistry reaction was developed with a 3,3'-diaminobenzidine (DAB) substrate kit (Vector Labs, Burlingame, CA, USA). Slides were then counterstained with hematoxylin for 5 min followed by several washes with ethanol (70%, 96%, 100%). Lastly, slides were dehydrated in xylene solutions and mounted with Di-N-butylPhthalate in Xylene solution (DPX, Sigma-Aldrich, Merck KGaA). Staining was evaluated in an Olympus BX41 TF microscope (Olympus, Tokyo, Japan).

**Tissue immunofluorescence**

Human and/or mouse skin and synovial membrane formalin-fixed paraffin-embedded sections were first incubated with 0.1% $NaBH_4$ to block non-specific autofluorescence. Slides were then incubated overnight at 4°C with primary antibodies against CD68 (KP1, 1/200), F4/80 (ADGRE1, EMR1; BM8, 1/100), MT-CO1 (PA1317-1, 1:100), phosphorylated-p65 (P-p65) (27.Ser 536, 1/100), or ZEB1 (HPA027524, 1/100) followed by incubation for 2 h at room temperature with their corresponding secondary antibodies, namely, Alexa Fluor® 488 Donkey Anti-Mouse (1/200), Alexa Fluor 488 Donkey Anti-Rabbit (1/500), Rhodamine X donkey anti-mouse (1/250) or Rhodamine X donkey anti-rabbit (1/500). Slides were then mounted with Prolong Gold® Antifade Reagent with DAPI (Thermo Fisher). Staining was evaluated in a Nikon Eclipse E600 microscope (Nikon, Tokyo, Japan), and images were processed with ImageJ software.

**Assessment of mitochondrial content and morphology by electron transmission microscopy**

*Zeb1*$^{WT}$ and *Zeb1*$^{\Delta M}$ mice were i.p. injected with PBS, LPS for the indicated periods or LPS + LPS (Supplementary Figure S1A). At least 0.5 x $10^6$ *Zeb1*$^{WT}$ and *Zeb1*$^{\Delta M}$ macrophages pooled from 3-6 mice of each genotype and condition were sorted by FACS and immediately put in freshly prepared ice-cold 3% glutaraldehyde solution in 0.1 M phosphate buffer. Sorted macrophages were then washed in PBS and fixed for 1 h in 500 µl of 3% glutaraldehyde in 0.1 M phosphate buffer at room temperature, centrifuged, and resuspended in 500 µl of fresh ice-cold 0.1 M phosphate buffer, and fixed for 72h at 4°C. Macrophages were then pelleted in Eppendorf tubes, washed in phosphate buffer, and incubated with 1% OsO4 for 90 min at 4°C. Samples were then dehydrated, embedded in Spurr's Low Viscosity embedding mixture (Electron Microscopy Sciences, Hatfield, PA, USA), and sectioned using Leica ultramicrotome (Leica Microsystems, Wetzlar, Germany). Ultra-thin sections (50-70 nm) were stained with 2% uranyl acetate for 10 min and with a lead-staining solution for 5min and observed using a JEOL JEM-1010 transmission electron microscope (JEOL Ltd., Akishima, Tokyo, Japan) coupled with an Orius SC1000 CCD camera (model 832) (Gatan Inc., Pleasanton, CA, USA) in the Electron Microscopy Unit at the University of Barcelona School of Medicine and Health Sciences (Barcelona, Spain). Image analysis and mitochondria quantification were conducted using ImageJ software. At least six independent images from three independent grids were analyzed from each genotype and condition.

**Oxygen consumption rate, spare respiratory capacity, and extracellular acidification rate**

Two x $10^5$ peritoneal cells were resuspended in DMEM without serum, seeded onto XF96 tissue culture plates (Agilent Technologies, Santa Clara, CA USA), and allowed to adhere for 1 h at room temperature in the absence of $CO_2$. Cells were then washed and then either left untreated or treated with LPS, MET+LPS, or LPS+LPS. To evaluate respiratory capacity, macrophages were incubated in XF DMEM medium with a pH of 7.4. The medium was supplemented with 10 mM glucose, 1 mM pyruvate, and 2 mM glutamine (Agilent Technologies). The plates were then incubated for 1 h at 37°C in a $CO_2$-free atmosphere. Oxygen consumption rate (OCR), extracellular acidification rate (ECAR), and spare respiratory capacity were measured using a Seahorse XF Cell Mito Stress Test Kit (Agilent Technologies) in a Seahorse XF96 Flux Analyzer (Agilent Technologies), following the manufacturer's instructions. Briefly, following three basal measurements each lasting 6 min, the different inhibitors of mitochondrial respiration were subsequently dispensed automatically, namely, 1.5 µM oligomycin, 0.5 FCCP, and 0.5 µM rotenone/Antimycin (Agilent Technologies) into plates. Total OCR was calculated as the average of the first three basal measurements, while spare respiratory capacity was determined by subtracting the basal OCR from the maximum OCR obtained after treating cells with FCCP.

**Assessment of mitochondrial protein translation**

Mitochondrial protein translation was assessed by Click-iT chemistry using a protocol modified from the ones described in (51,52) Briefly, 1 x $10^6$ peritoneal cells from a pool of either three *Zeb1*$^{WT}$ mice or three *Zeb1*$^{\Delta M}$ mice were plated in non-adherent 6-well. Cells were incubated in DMEM with high glucose (4.5 g/L) but without glutamine, methionine, and cystine (Thermo Fisher) supplemented with 10% FBS, 1% penicillin, streptomycin (Lonza), 4 mM L-glutamine (Lonza), 30 mg/L L-Cystine (Thermo Fisher), 1 mM sodium pyruvate (Lonza). Then, cells were either subjected to one (acute inflammation) or two (immunosuppression) doses of 100

ng/ml of LPS as follows. First, cells were left untreated (for the untreated and acute inflammation) or treated with 100 ng/ml LPS (cells in the LPS tolerance condition) for 3 hr. All cells received 2 ml of fresh DMEM and were incubated for 48 h. At that time, cells were washed with PBS and incubated for 45 min with complete DMEM without methionine with 100 ng/ml of LPS (acute inflammation and immunosuppression) or left untreated. Wherever indicated, cells were treated for 20 min with 100 µg/ml of emetine dihydrochloride hydrate (Sigma-Aldrich) to inhibit cytosolic protein translation and/or 10 µg/ml of doxycycline hydrochloride (Fisher Bioreagents, ThermoFisher Scientific) to inhibit mitochondrial translation. Next, cells were treated for 1 h with 750 µM of L-homopropargylglycine (L-HPG, Jena Biosciences). Cells were then washed with PBS, stained for 30 min with Alexa Fluor® 488-conjugated F4/80 antibody at 4ºC, and fixed for 20 min with 1% paraformaldehyde at room temperature. Lastly, cells were permeabilized and stained with Click-iT™ Alexa Fluor™ 647 Flow Cytometry Assay Kit (Thermo Fisher Scientific) as per the manufacturer's instructions. L-HPG incorporation was quantified by FACS. Cells that had been treated with emetine dihydrochloride hydrate or doxycycline were used as baseline controls for cytosolic and mitochondrial translation, respectively.

## SUPPLEMENTARY REFERENCES


1. Guo Y, et al. Zeb1 induces immune checkpoints to form an immunosuppressive envelope around invading cancer cells. Sci Adv. **7**, eabd7455 (2021).
2. Braga D, et al. A longitudinal study highlights shared aspects of the transcriptomic response to cardiogenic and septic shock. *Crit Care*. **23**, 41 (2019).
3. Soberanes S, et al. Metformin Targets Mitochondrial Electron Transport to Reduce Air-Pollution-Induced Thrombosis. *Cell Metab.* **29**, 335-347 (2019).
4. Yao Y, et al. Type I interferon: potential therapeutic target for psoriasis? *PLoS One* **3**, e2737 (2008).
5. Rosenberg A, et al. Divergent gene activation in peripheral blood and tissues of patients with rheumatoid arthritis, psoriatic arthritis and psoriasis following infliximab therapy. *PLoS One.* **9**, e110657. (2014)
6. Severino P, et al. Patterns of gene expression in peripheral blood mononuclear cells and outcomes from patients with sepsis secondary to community acquired pneumonia. *PLoS One* **25**, 9:e91886 (2014).
7. Bar EE, Chaudhry A, Farah MH, Eberhart CG. Hedgehog signaling promotes medulloblastoma survival via BclII. *Am J Pathol* **170**, 347-355 (2007).
8. Štorcelová M, Vicián M, Reis R, Zeman M, Herichová I. Expression of cell cycle regulatory factors hus1, gadd45a, rb1, cdkn2a and mre11a correlates with expression of clock gene per2 in human colorectal carcinoma tissue. *Mol Biol Rep.* **40**, 6351-61 (2013).
9. Lechner MG, Liebertz DJ, Epstein AL. Characterization of cytokine-induced myeloid-derived suppressor cells from normal human peripheral blood mononuclear cells. *J Immunol*. **185**, 2273-84 (2010).
10. Hoetzenecker W, et al. ROS-induced ATF3 causes susceptibility to secondary infections during sepsis-associated immunosuppression. Nat Med. 18;18:128-34 (2011).
11. Pate KT, et al. Wnt signaling directs a metabolic program of glycolysis and angiogenesis in colon cancer. *EMBO J.* **33**, 1454-73 (2014).
12. Yoon BR, et al. Role of SLC7A5 in Metabolic Reprogramming of Human Monocyte/Macrophage Immune Responses. *Front Immunol*. **9**, 53 (2018).
13. Huang H, et al. Upregulation of SQSTM1/p62 contributes to nickel-induced malignant transformation of human bronchial epithelial cells. *Autophagy*. **12**, 1687-1703 (2016).

14. Peña C, et al.F. E-cadherin and vitamin D receptor regulation by SNAIL and ZEB1 in colon cancer: clinicopathological correlations. *Human Mol Gen* **14**, 3361-70 (2005).
15. Campagnolo P, et al. Resveratrol-induced vascular progenitor differentiation towards endothelial lineage via MiR-21/Akt/β-catenin is protective in vessel graft models. *PloS One*. **10**, e0125122 (2015).
16. Esteras N, et al. Altered cell cycle-related gene expression in brain and lymphocytes from a transgenic mouse model of Alzheimer's disease [amyloid precursor protein/presenilin 1 (PS1)]. *Eur J Neurosci*. **36**, 2609-18 (2012).
17. Cho S, Wu CJ, et al. miR-23~27~24 clusters control effector T cell differentiation and function. *J Exp Med*. **213**, 235-49 (2016).
18. Matyi S, et al. The effect of different levels of dietary restriction on glucose homeostasis and metabolic memory. *Geroscience*. **40**, 139-149 (2018).
19. Komakula SSB, et al. The DNA Repair Protein OGG1 Protects Against Obesity by Altering Mitochondrial Energetics in White Adipose Tissue. *Sci Rep* **8**, 14886 (2018).
20. El-Merhie N, et al. Differential Alterations of the Mitochondrial Morphology and Respiratory Chain Complexes during Postnatal Development of the Mouse Lung. *Oxid Med Cell Longev*. 9169146 (2017).
21. Bhutia YD, Babu E, Ganapathy V. Interferon-γ induces a tryptophan-selective amino acid transporter in human colonic epithelial cells and mouse dendritic cells. *Biochim Biophys Acta*. **1848**, 453-62 (2015).
22. Duran A, et al. p62/SQSTM1 by Binding to Vitamin D Receptor Inhibits Hepatic Stellate Cell Activity, Fibrosis, and Liver Cancer. *Cancer Cell.* **30**, 595-609 (2016).
23. Brabletz S, et al. Generation and characterization of mice for conditional inactivation of Zeb1. *Genesis*. **55**, e23024. (2017).
24. Wei FY, et al. Cdk5rap1-mediated 2-methylthio modification of mitochondrial tRNAs governs protein translation and contributes to myopathy in mice and humans. *Cell Metab*. **21**, 428-442 (2015).
25. Singer M, et al. The Third International Consensus Definitions for Sepsis and Septic Shock (Sepsis-3). *JAMA* **315**, 801-810 (2016).
26. Knaus WA, Draper EA, Wagner DP, Zimmerman. JEAPACHE II: a severity of disease classification system. *Crit Care Med*. **13**, 818-29 (1985).

27. Vincent JL, et al. The SOFA (Sepsis-related Organ Failure Assessment) score to describe organ dysfunction/failure. On behalf of the Working Group on Sepsis-Related Problems of the European Society of Intensive Care Medicine. *Intensive Care Med*. **22**, 707-710 (1996).
28. Taylor W, Gladman D, Helliwell P, Marchesoni A, Mease P, Mielants H. Classification criteria for psoriatic arthritis: development of new criteria from a large international study. *Arthritis & Rheum*. **54**, 2665-73 (2006).
29. Schoels M, Aletaha D, Funovits J, Kavanaugh A, Baker D, Smolen JS. Application of the DAREA/DAPSA score for assessment of disease activity in psoriatic arthritis. *Annals Rheum Dis*. **69**, 1441-7 (2010).
30. Schulthess J, et al. The Short Chain Fatty Acid Butyrate Imprints an Antimicrobial Program in Macrophages. *Immunity* **50**, 432-445.e7 (2019).
31. Cong L, et al. Multiplex genome engineering using CRISPR/Cas systems. *Science*. **339**, 819-823 (2013).
32. Yang H, Wang H, Jaenisch R. Generating genetically modified mice using CRISPR/Cas-mediated genome engineering. *Nat Protoc*. **9,**1956-1968 (2014).
33. Behringer R, Gertsenstein M, Vintersten Nagy K, Nagy A. *Manipulating the mouse embryo: A laboratory manual*, 2nd edition. Cold Spring Harbor, New York: Cold Spring Harbor Laboratory Press (2014).
34. Clausen BE, Burkhardt C, Reith W, Renkawitz R, Forster I. Conditional gene targeting in macrophages and granulocytes using LysMcre mice. *Transgenic Res* **8**, 265-277 (1999).
35. Kang S, et al. Semaphorin 6D reverse signaling controls macrophage lipid metabolism and anti-inflammatory polarization. *Nat Immunol*. **19**, 561-570 (2018).
36. Torretta S, et al. D-mannose suppresses macrophage IL-1β production. *Nat Commun*. **11**, 6343 (2020).
37. Mogilenko DA, et al. Metabolic and innate immune cues merge into a specific inflammatory response via the UPR. *Cell*. **177**, 1201-1216 (2019).
38. van der Fits, L., et al. Imiquimod-induced psoriasis-like skin inflammation in mice is mediated via the IL-23/IL-17 axis. *J. Immunol.* **182**, 5836-5845 (2009).
39. Zhang X, Goncalves R, Mosser DM. The isolation and characterization of murine macrophages. *Curr Protoc Immunol.* **83**,14.1.1-14.1.14 (2008).
40. Keiran N, et al. SUCNR1 controls an anti-inflammatory program in macrophages to regulate the metabolic response to obesity. *Nat Immunol*. **20**, 581-592 (2019).

41. Sánchez-Tilló E, de Barrios O, Siles L, Cuatrecasas M, Castells A, Postigo A. β-catenin/TCF4 complex induces the epithelial-to-mesenchymal transition (EMT)-activator ZEB1 to regulate tumor invasiveness. *Proc. Natl. Acad. Sci. USA*. **108**, 19204-9 (2011).
42. Dobin A, et al. STAR: ultrafast universal RNA-seq aligner. *Bioinformatics*. **29**,15-21 (2013).
43. Li B, Dewey CN. RSEM: accurate transcript quantification from RNA-Seq data with or without a reference genome. *BMC Bioinformatics* **12**, 323 (2011).
44. Love MI, Huber W, Anders S. Moderated estimation of fold change and dispersion for RNA-seq data with DESeq2. *Genome Biol*. **15**, 5508 (2014).
45. Raudvere U, et al. g: Profiler: a web server for functional enrichment analysis and conversions of gene lists (2019 update). *Nucleic Acids Res*. **47**, W191-8 (2019).
46. Mootha VK, et al. PGC-1α-responsive genes involved in oxidative phosphorylation are coordinately downregulated in human diabetes. *Nat Genet*. **34**, 267-73 (2003).
47. Subramanian A, et al. Gene set enrichment analysis: a knowledge-based approach for interpreting genome-wide expression profiles. *Proc Nat Acad Sci USA*. **102**, 15545-50 (2005).
48. Shannon P, et al. Cytoscape: a software environment for integrated models of biomolecular interaction networks. *Genome Res.* **13**, 2498-504 (2003) .
49. Yu, LG Wang, Y Han, QY He. clusterProfiler: an R package for comparing biological themes among gene clusters. *OMICS* **16**, 284-287 (2012).
50. Xie, P et al. Quantitative PCR measurement of tRNA 2-methylthio modification for assessing type 2 diabetes risk. *Clin. Chem*. **59**,1604-1612 (2013)
51. Zhang X, et al. MicroRNA directly enhances mitochondrial translation during muscle differentiation. *Cell* **158**, 607-619 (2014).
52. Liu, F., et al. ALKBH1-Mediated tRNA Demethylation Regulates Translation. *Cell* **167**, 816-828.e16 (2016).
