# Supplementary material for: Inflammatory macrophages reprogram to immunosuppression by reducing mitochondrial translation
Source: Nat Commun. 2023 Nov 17;14:7471. doi: 10.1038/s41467-023-42277-4 (PMC10656499; doi:10.1038/s41467-023-42277-4)
Supplement: Supplementary file 3 — Reporting Summary [file 41467_2023_42277_MOESM3_ESM.pdf]

nature portfolio

Corresponding author(s): Antonio POSTIGO & Marlies CORTES

Last updated by author(s): 2023/09/19

Reporting Summary

Nature Portfolio wishes to improve the reproducibility of the work that we publish. This form provides structure for consistency and transparency in reporting. For further information on Nature Portfolio policies, see our Editorial Policies and the Editorial Policy Checklist.

Statistics

For all statistical analyses, confirm that the following items are present in the figure legend, table legend, main text, or Methods section.

n/a	Confirmed	
☐	☒	The exact sample size (*n*) for each experimental group/condition, given as a discrete number and unit of measurement
☐	☒	A statement on whether measurements were taken from distinct samples or whether the same sample was measured repeatedly
☐	☒	The statistical test(s) used AND whether they are one- or two-sided<br>*Only common tests should be described solely by name; describe more complex techniques in the Methods section.*
☒	☐	A description of all covariates tested
☒	☐	A description of any assumptions or corrections, such as tests of normality and adjustment for multiple comparisons
☐	☒	A full description of the statistical parameters including central tendency (e.g. means) or other basic estimates (e.g. regression coefficient) AND variation (e.g. standard deviation) or associated estimates of uncertainty (e.g. confidence intervals)
☐	☒	For null hypothesis testing, the test statistic (e.g. *F*, *t*, *r*) with confidence intervals, effect sizes, degrees of freedom and *P* value noted<br>*Give P values as exact values whenever suitable.*
☒	☐	For Bayesian analysis, information on the choice of priors and Markov chain Monte Carlo settings
☒	☐	For hierarchical and complex designs, identification of the appropriate level for tests and full reporting of outcomes
☒	☐	Estimates of effect sizes (e.g. Cohen's *d*, Pearson's *r*), indicating how they were calculated

Our web collection on statistics for biologists contains articles on many of the points above.

Software and code

Policy information about availability of computer code

Data collection	FACS data were collected using FACSDiva Software Version 6.1.<br>qPCR data was collected using LightCycler® 96 Application Software Version 1.1 (Roche).
Data analysis	GraphPad Prism 9.3.1 was used for statical analysis., ImageJ 2.1.0 for IF composite images and Western blot intensity analysis., FlowJo 10.8.1 for FACS data analysis., GSEA v4.3.2 for GSEA analysis., MetaboAnalystR 3.0 to generate heatmaps., GainData® Arigo's ELISA calculator., Cytoscape 3.9.1 to generate cnetplot network hub., and Real-Time PCR LightCycler® 96 Application Software Version 1.1 (Roche) software was used to analyze qPCR data.

For manuscripts utilizing custom algorithms or software that are central to the research but not yet described in published literature, software must be made available to editors and reviewers. We strongly encourage code deposition in a community repository (e.g. GitHub). See the Nature Portfolio guidelines for submitting code & software for further information.

Data

Policy information about availability of data

All manuscripts must include a data availability statement. This statement should provide the following information, where applicable:

- Accession codes, unique identifiers, or web links for publicly available datasets
- A description of any restrictions on data availability
- For clinical datasets or third party data, please ensure that the statement adheres to our policy

Data generated in this study are provided in the Source Data file. The RNA-seq data generated in the study were uploaded on the Gene Expression Omnibus (GEO) database under the accession code GSE207328 with link: https://www.ncbi.nlm.nih.gov/geo/query/acc.cgi?acc=GSE207328. Reads in the RNAseq were mapped against the latest mouse reference genome assembly (GRCm38), which is available at the NIH GenBank repository website with link https://www.ncbi.nlm.nih.gov/datasets/genome/GCF_000001635.20/

Datasets from RNA-seq published elsewhere were retrieved from the Gene Expression Omnibus (GEO) database with accession codes GSE131411; GSE14905; GSE57383; GSE48080 through the following public domain resources:
https://www.ncbi.nlm.nih.gov/geo/query/acc.cgi?acc=GSE131411
https://www.ncbi.nlm.nih.gov/geo/query/acc.cgi?acc=GSE14905
https://www.ncbi.nlm.nih.gov/geo/query/acc.cgi?acc=GSE57383
https://www.ncbi.nlm.nih.gov/geo/query/acc.cgi?acc=GSE48080

Research involving human participants, their data, or biological material

Policy information about studies with human participants or human data. See also policy information about sex, gender (identity/presentation), and sexual orientation and race, ethnicity and racism.

Reporting on sex and gender	The sex of patients is detailed in Supplementary Tables S1, S2 and S3. As controls, we included 13 healthy volunteers, 10 females and 3 males. For septic patients, we recruited 10 males and 10 females and for psoriatic arthritis (PsA) patients, 7 males and 5 females.
Reporting on race, ethnicity, or other socially relevant groupings	We did not collect data on race, ethnicity, and socioeconomic status in this study.
Population characteristics	The population characteristics of Healthy volunteers, and septic and PsA patients are detailed in Supplementary Tables S1, S2 and S3.
Recruitment	Septic patients were admitted to the Medical Intensive Care Unit (Hospital Clinic of Barcelona, Spain) within the first 24 hours of sepsis or septic shock and classified as sepsis or septic shock according to the criteria established by the United States' Society of Critical Care Medicine and the European Society of Intensive Care Medicine third consensus conference definitions (2016, Sepsis-3) (Seymour et al., 2016). The exclusion criteria were: pregnancy, patients with active onco-hematological disease, infection with the human immunodeficiency and/or hepatitis C viruses, inclusion in a clinical trial in the previous month, patients with life expectancy less than 6 months, with “Do Not Resuscitate” establishment orders or futile care prior to enrollment, and patients with a septic shock derived from an unresolved surgical problem. The severity of illness and organ dysfunction were assessed with the Acute Physiologic And Chronic Health Evaluation (APACHE)-II and the Sequential Organ Failure Assessment (SOFA) scores (Minne et al., 2008).<br><br>Psoriatic arthritis (PsA) patients, aged 26.9 to 72.7 (see Supplementary Table S2) under clinical control at the Arthritis Unit, Department of Rheumatology (Hospital Clinic, Barcelona, Spain) were diagnosed according to the Classification Criteria for Psoriatic Arthritis (CASPAR) criteria established by the joint criteria established by the American College of Rheumatology and European League Against Rheumatism (Taylor et al., 2006). Patients were in active disease according to Disease Activity Score using 28 joint counts (DAS28) and Disease Activity Index for Psoriatic Arthritis (DAPSA) scores (Schoels et al., 2010). Peripheral blood and synovial membrane from these patients were matched with blood samples from healthy controls and synovial membrane from osteoarthritis patients according to classification criteria. Skin samples of psoriatic and melanoma patients were obtained from the Dept. of Pathology at Hospital Clínico San Carlos (Madrid, Spain).
Ethics oversight	The use of human samples in this study was approved by the local Institutional Review Board, namely the Clinical Experimentation Ethics Committee, Drug Research Ethics Committee at Hospital Clínic of Barcelona (Barcelona, Spain) (https://t.ly/yTMSr). The Committee approved the use of human samples (septic patients, PsA patients, healthy controls) in the study under protocols references HCB/2017/0767, HCB/2019/1012, and HCB/2020/0100. All human samples were obtained with the informed consent of patients and donors, and conformed with the principles of the Helsinki Declaration.

Note that full information on the approval of the study protocol must also be provided in the manuscript.

Field-specific reporting

Please select the one below that is the best fit for your research. If you are not sure, read the appropriate sections before making your selection.

☒ Life sciences ☐ Behavioural & social sciences ☐ Ecological, evolutionary & environmental sciences

For a reference copy of the document with all sections, see nature.com/documents/nr-reporting-summary-flat.pdf

Life sciences study design

All studies must disclose on these points even when the disclosure is negative.

Sample size	No prior sample size calculation was performed. Sample sizes were chosen guided by prior knowledge, and ethical considerations, and based on previous studies (Nat Immunol 19:561-570; Nat Commun 11:6343). We maximized the use of animals while adhering to ethical principles and the 3 Rs (replacement, reduction, refinement) to minimize the number of animals employed. Group distribution was carefully balanced, with fewer mice allocated to the "untreated" condition, a decision informed by the well-established fact that Zeb1WT and Zeb1ΔM macrophages typically do not display distinct characteristics under basal, untreated conditions and the study is focused on inflammatory process response. To ensure robust statistical power, we analyzed 3 to 15 mice per group in our in vivo assays, carefully calibrating this range to detect meaningful differences. In vitro assays tend to have lower variability, so we employed fewer biological replicates, here all experiments were conducted with a minimum of 3 to 4 samples from mice. In human experiments, sample sizes were adjusted to achieve adequate statistical power, considering human population variability. Sample sizes were explicitly detailed in figure legends and represented as data points in graphs. Each experiment was replicated at least twice, bolstering the robustness of our findings.
Data exclusions	No data were excluded from the study.
Replication	See Methods and the figure legends for detailed information on the number of replicates in each experiment. Experiments were repeated at least twice with consistent results or data pooled from 2-3 biological replicates for each independent experiment.
Randomization	For all animal studies, mice of each genotype were age- and sex-matched and randomly assigned before the challenge with either vehicle or treatment, and they were processed in parallel. To minimize cage effects, mice were housed in mixed cages.<br><br>In the case of in vitro experiments, macrophages from age- and sex-matched mice of each genotype were isolated and randomly assigned to either treatment or control groups.
Blinding	In the RNAseq analyses, the experiments were blinded, as the analysis was conducted by specialists who did not know the identity of the samples. For the remaining experiments, blinding was not technically feasible as the transgenic mice were cohoused and thus had to be marked for identification. Additionally, throughout the study, all data collection and analysis were quantitative rather than qualitative in nature.

Reporting for specific materials, systems and methods

We require information from authors about some types of materials, experimental systems and methods used in many studies. Here, indicate whether each material, system or method listed is relevant to your study. If you are not sure if a list item applies to your research, read the appropriate section before selecting a response.

Materials & experimental systems

n/a	Involved in the study	
☐	☒	Antibodies
☒	☐	Eukaryotic cell lines
☒	☐	Palaeontology and archaeology
☐	☒	Animals and other organisms
☒	☐	Clinical data
☒	☐	Dual use research of concern
☒	☐	Plants

Methods

n/a	Involved in the study	
☒	☐	ChIP-seq
☐	☒	Flow cytometry
☒	☐	MRI-based neuroimaging

Antibodies

Antibodies used

Primary and secondary antibodies used in the study are listed in Supplementary Table S5

PRIMARY UNCONJUGATED ANTIBODIES

B-actin Santa Cruz Biotechnology C4 (sc-47778)
ADGRE1 (F4/80) / EMR1 Santa Cruz Biotechnology BM8 (sc-52664)
Arginase I Santa Cruz Biotechnology V-20 (sc-18354)
CD68 Santa Cruz Biotechnology KP1 (sc-20060)
GAPDH Cell Signaling 14C10 (2118L)
GAPDH Proteintech 1E6D9 (60004-1-Ig)
IL6 ImmunoTools GmbH IT1G2 (22450061)
IL6 R&D Systems 6708 (MAB206)
MT-CO1 Booster Biological Technology PA1317-1
p62 (SQSTM1). Santa Cruz Biotechnology D-3 (sc-28359)
p70(S6K) Proteintech 14485-1-AP
Phosphorylated p65 (P-p65) Santa Cruz Biotechnology 27.Ser 536 (sc-136548)
Phospho-p70(S6K) (Thr389) Proteintech 28735-1-AP
SLC7A8 (LAT2) ImmunoGlobe (20180105)

SLC7A8 (LAT2) Origene OTI5A9
TOMM20 Abcam plc ab56783
ZEB1 Santa Cruz Biotechnology H-3 (sc-515797)
ZEB1 Home-made (Sci Adv. 7, eabd7455 (2021)) N/A
ZEB1 Sigma-Aldrich AMAb90510
ZEB1 Sigma-Aldrich HPA027524

CONJUGATED ANTIBODIES

CD11b: anti-mouse CD11b PE-conjugated ImmunoTools GmbH M1/70.15 (22159114)
CD45: anti-mouse CD45-conjugated PerCP/Cy5.5 BioLegend 30-F11 (103132)
CD68: Alexa Fluor® 488 anti-human/mouse/rat CD68 Ab Mouse IgG1 (kappa light chain) Santa Cruz Biotechnology KP1 (sc-20060-AF488)
F4/80: APC anti-mouse mAb. Rat IgG2a, κ Biolegend BM8 (123116)
F4/80: Alexa Fluor® 488 anti-mouse F4/80. Rat IgG2a, κ BioLegend BM8 (123119)
CD11c: Anti-CD11c Armenian Hamster Monoclonal Antibody (PE (Phycoerythrin)/Cy7®) [clone: N418]
Ly-6C: Ly-6C Monoclonal Antibody (HK1.4), PerCP-Cyanine5.5, eBioscience™
GR-1: FITC- conjugated monoclonal antibody RB6-8C5 to mouse Gr-1
CD14: anti-human CD14 PerCP-conjugated ImmunoTools GmbH 18D11 (21620145)
IL6: Anti-IL-6 Rat Monoclonal Antibody (PE (Phycoerythrin)) [clone: MP5-20F3]

Dilution for the used antibodies are specified in the manuscript where applicable.

Validation

The antibodies used in the study were validated by the corresponding manufacturer and/or were widely used in the literature. Commercial antibodies were employed in accordance with the manufacturers' recommendations as provided on their datasheets and official websites.

Antibodies for Immunoblotting
B-actin Santa Cruz Biotechnology C4 (sc-47778) https://www.scbt.com/p/beta-actin-antibody-c4 (13555 citations)
Arginase I Santa Cruz Biotechnology V-20 (sc-18354) https://www.scbt.com/p/arginase-i-antibody-v-20 (12 citations)
GAPDH Cell Signaling 14C10 (2118L) https://www.cellsignal.com/products/primary-antibodies/gapdh-14c10-rabbit-mab/2118?_requestid=2169594 (7410 citations)
GAPDH Proteintech 1E6D9 (60004-1-Ig) https://www.ptglab.com/products/GAPDH-Antibody-60004-1-Ig.htm#product-information (8298 citations)
IL6 ImmunoTools GmbH IT1G2 (22450061) http://www.immunotools.de/html/datas-m/22450061.pdf
IL6 R&D Systems 6708 (MAB206) https://www.rndsystems.com/products/human-primate-il-6-antibody-6708_mab206 (77 citations)
p62 (SQSTM1). Santa Cruz Biotechnology D-3 (sc-28359) https://www.scbt.com/p/sqstm1-antibody-d-3 (560 citations)
p70(S6K) Proteintech 14485-1-AP https://www.ptglab.com/products/p70(S6K)-Antibody-14485-1-AP.htm (135 citations)
Phospho-p70(S6K) (Thr389) Proteintech 28735-1-AP https://www.ptglab.com/products/Phospho-p70-S6K-Thr389-Antibody-28735-1-AP.htm (11 citations)
SLC7A8 (LAT-2) ImmunoGlobe (0142-10) https://www.immunoglobe.com/antibodies/items/slc7a8.html (3 citations)
SLC7A8 (LAT2) Origene OTI5A9 (TA500503) https://www.origene.com/catalog/antibodies/primary-antibodies/ta500503/lat2-slc7a8-mouse-monoclonal-antibody-clone-id-oti5a9 (1 citation)
TOMM20 Abcam plc ab56783 https://www.abcam.com/products/primary-antibodies/tomm20-antibody-4f3-bsa-and-azide-free-ab56783.html (198 citations)
ZEB1 Home-made (Sci Adv. 7, eabd7455 (2021))

Antibody for ChiP
ZEB1 Santa Cruz Biotechnology H-3 (sc-515797) https://www.scbt.com/p/zeb1-antibody-h-3 (37 citations)

Antibodies for Immunoblotting and Immunostaining
MT-CO1 Booster Biological Technology PA1317-1 https://www.bosterbio.com/anti-mtco1-antibody-pa1317-1-boster.html
Phosphorylated p65 (P-p65) Santa Cruz Biotechnology 27.Ser 536 (sc-136548) https://www.scbt.com/p/p-nfkappab-p65-antibody-27-ser-536 (254 citations)
ZEB1 Home-made (Sci Adv. 7, eabd7455 (2021))
ZEB1 Sigma-Aldrich HPA027524 https://www.sigmaaldrich.com/ES/es/product/sigma/hpa027524 (120 citations)

Antibodies for Immunostaining
ADGRE1 (F4/80) / EMR1 Santa Cruz Biotechnology BM8 (sc-52664) https://www.scbt.com/p/f4-80-antibody-bm8 (98 citations)
CD68 Santa Cruz Biotechnology KP1 (sc-20060) https://www.scbt.com/p/cd68-antibody-kp1 (201 citations)
CD68: Alexa Fluor® 488 anti-human/mouse/rat CD68 Ab Mouse IgG1 (kappa light chain) Santa Cruz Biotechnology KP1 (sc-20060-AF488) https://www.scbt.com/p/cd68-antibody-kp1 (201 citations)
ZEB1 Sigma-Aldrich AMAb90510 https://www.sigmaaldrich.com/ES/es/product/sigma/amab90510 (14 citations)

Antibodies for FACS
CD11b: anti-mouse CD11b PE-conjugated ImmunoTools GmbH M1/70.15 (22159114) http://www.immunotools.de/html/datas-pe-m/22159114.pdf (3 citations)
CD45: anti-mouse CD45-conjugated PerCP/Cy5.5 BioLegend 30-F11 (103132) https://www.biolegend.com/fr-lu/products/percp-cyanine5-5-anti-mouse-cd45-antibody-4264?GroupID=BLG6829 (214 citations)
F4/80: APC anti-mouse mAb. Rat IgG2a, κ Biolegend BM8 (123116) https://www.biolegend.com/de-de/explore-new-products/apc-anti-mouse-f4-80-antibody-4071 (402 citations)
F4/80: Alexa Fluor® 488 anti-mouse F4/80. Rat IgG2a, κ BioLegend BM8 (123119) https://www.biolegend.com/en-us/products/alexa-fluor-488-anti-mouse-f4-80-antibody-4073?GroupID=BLG5319
CD11c: Anti-CD11c Armenian Hamster Monoclonal Antibody (PE (Phycoerythrin)/Cy7®) [clone: N418] https://www.biolegend.com/en-us/products/pe-cyanine7-anti-mouse-cd11c-antibody-3086?GroupID=BLG11937 (225 citations)

Ly-6C: Ly-6C Monoclonal Antibody (HK1.4), PerCP-Cyanine5.5, eBioscience™ https://www.thermofisher.com/antibody/product/Ly-6C-Antibody-clone-HK1-4-Monoclonal/45-5932-82 (65 citations)
GR-1: FITC- conjugated monoclonal antibody RB6-8C5 to mouse Gr-1 https://www.biolegend.com/fr-ch/products/fitc-anti-mouse-ly-6g-ly-6c-gr-1-antibody-458?GroupID=BLG4876 (127 citations)
CD14: anti-human CD14 PerCP-conjugated ImmunoTools GmbH 18D11 (21620145) http://www.immunotools.de/html/datas-percp/21620145.pdf (1 citation)
IL6: Anti-IL-6 Rat Monoclonal Antibody (PE (Phycoerythrin)) [clone: MP5-20F3] https://www.biolegend.com/en-us/products/pe-anti-mouse-il-6-antibody-972?GroupID=GROUP24 (19 citations)

Animals and other research organisms

Policy information about studies involving animals; ARRIVE guidelines recommended for reporting animal research, and Sex and Gender in Research

Laboratory animals	The conditional Zeb1 flox allele mouse (Zeb1fl/fl, herein referred as Zeb1WT) (mouse model reference: B6.B6CBA-Zeb1em1/cnbbm) was generated in the joint National Biotechnology Center (CSIC-CNB) / Severo Ochoa's Molecular Biology Center (CSIC-CBMSO) Transgenesis Unit at the Spanish National Research Council (CSIC) and Autonomous University of Madrid (Madrid, Spain). Mice were crossed multiple times with twild type C57BL6/Jcrl mice to generate the Zeb1fl/+ mice. Zeb1fl/fl (Zeb1WT) mouse with the presence of correct LoxP sequences was then crossed with a mouse carrying the Cre recombinase selectively in myeloid cells under the control of the endogenous lysozyme 2 (Lyz2, also referred as LysM) promoter/enhancer (official name: B6.129P2-Lyz2tm1(cre)Ifo/J). The sex of mice is indicated in Supplementary Methods. As noted in the original manuscript, the experimental mouse model of LPS-induced septic shock was conducted in 8-10 week-old female mice, which have been shown to have a higher number of peritoneal macrophages (Blood, 118:5918-5927). For ex vivo experiments, macrophages were isolated from 6-10 week-old female mice. For the IMQ-induced mouse model of psoriasis, 10-12 week-old male mice were used. When we used and compared both male and female mice in the same experiments (e.g., in vivo determination of ROS, Supplementary Figure S5E), we found no difference between both sexes. Mice were 6-12 weeks old with average weight of 20-25g. All mice were maintained in animal facility of the Faculty of medicine (University of Barcelona) and housed in a temperature-controlled room of 21–22 °C with a 12-hour light-dark cycle and were fed with standard rodent chow and given water ad libitum. All mice were euthanized by cervical dislocation.
Wild animals	No wild animals were used in the study.
Reporting on sex	The sex of mice used in the sepsis and IMQ models is reported in Methods. Females were used for LPS acute inflammation and immunossuppression. Males were used for IMQ psoriasis model.
Field-collected samples	Study did not involve field samples.
Ethics oversight	The use of mouse models in the study was approved by and experiments were conducted in accordance with the guidelines established by the Animal Experimental Committee at the University of Barcelona School of Medicine (Barcelona, Spain) and by the Generalitat (Government of Catalonia) under references 396/18 and 133/19 and 1041, respectively.

Note that full information on the approval of the study protocol must also be provided in the manuscript.

Flow Cytometry

Plots

Confirm that:

- [x] The axis labels state the marker and fluorochrome used (e.g. CD4-FITC).
- [x] The axis scales are clearly visible. Include numbers along axes only for bottom left plot of group (a 'group' is an analysis of identical markers).
- [x] All plots are contour plots with outliers or pseudocolor plots.
- [x] A numerical value for number of cells or percentage (with statistics) is provided.

Methodology

Sample preparation	The preparation of samples is described in the Methods section. Cells were suspended in FACS buffer (2% FBS, 1 mM EDTA in PBS) with the corresponding antibodies, and incubated for 30-60 min at 4ºC. Samples were then washed and resuspended in FACS buffer.
Instrument	FACSCanto™ II analyzer, BD LSRFortessa™ Cell Analyzer (BD Biosciences, San Jose, CA, USA)
Software	FlowJo 10.8.1 for Mac
Cell population abundance	At least 10,000 cells were collected for each experiment.
Gating strategy	The gating strategy is described in methods and shown in Supplementary Fig S1M

- [x] Tick this box to confirm that a figure exemplifying the gating strategy is provided in the Supplementary Information.
